# The Organism as a System: Insights from Paul Weiss

**DOI:** 10.1007/s10441-026-09539-2

**Published:** 2026-10-22

**Authors:** Daniel J. Nicholson

**Affiliations:** Department of Philosophy, George Mason University, 4400 University Drive, Fairfax, VA 22030 USA

**Keywords:** Systems thinking, Organicism, Reductionism, Genocentrism, Determinism, Mechanicism

## Abstract

Despite the remarkable successes of molecular biology over the past three quarters of a century, there is growing recognition that organisms must be understood as integrated systems if we are to fully grasp their most distinctive features. Yet considerable uncertainty remains (and, in some quarters, outright skepticism) about how ‘systems thinking’ can actually deepen our understanding of organisms—beyond simply reminding us of such familiar maxims as ‘the whole is more than the sum of its parts’. In this paper, I draw on the largely forgotten organicist ideas of the experimental and theoretical biologist Paul Weiss (1898–1989) to outline a workable, intuitive, and non-formal systems-oriented conception of the organism, and proceed to illustrate its value and effectiveness across a range of explanatory contexts. Specifically, I argue that a proper understanding of the organism as a system—lucidly anticipated by Weiss more than half a century ago—provides the necessary conceptual resources to diagnose and overcome the problems with four prominent doctrines that remain deeply entrenched in contemporary biology: reductionism, genocentrism, determinism, and mechanicism.

[T]here is no phenomenon in a living system that is *not* molecular, but there is none that is *only* molecular, either. It is one thing not to see the forest for the trees, but then to go on to deny the reality of the forest is a more serious matter; for it is not just a case of myopia, but one of self-inflicted blindness.

— Paul Weiss (1969a: 10–11)

## Introduction

Twenty-first century biology has been guided by the realization that the grand molecular revolution of the last century has largely run its course, and that sooner or later we will need to integrate our findings and complement them with different theoretical perspectives—particularly, holistic or *systemic* ones (e.g., Rose 1998; Woese 2004; Noble 2006; Ball 2023; Rosslenbroich 2023). The problem is that nobody seems to agree on what a ‘system’ actually is in biology, let alone what it means to treat the organism as one.

Of course, the field of *systems biology* has developed enormously over the past twenty-five years. However, it is not clear that it offers an understanding of cells and organisms that is all that different from the one it inherited from twentieth-century molecular biology. The recognition that we need to move beyond characterizing individual parts (such as genes and proteins) is certainly there, but what systems biology offers in its place is not really a concern with ‘the whole’, but rather with how the parts interact with one another in increasing levels of complexity. If there *is* a whole, conceptually speaking, it is perpetually on the horizon—to be eventually arrived at piecemeal through the progressive mapping of the relations between the parts.

Most systems biologists understand the term ‘system’ pragmatically, rather than theoretically, as a vague but convenient label that can be applied to any network of biological components that we aim to apprehend by means of high-throughput technologies and computational tools (O’Malley and Dupré 2005; Krohs and Callebaut 2007). The large-scale implementation of these novel methodologies has enabled massive datasets of ‘part lists’ to be generated and collectively analysed, resulting in the proliferation of predictive mathematical models and in silico simulations of system-level behaviours that seek to infer causal relations from correlations identified in the data. Despite these innovations, though, systems biology has proven to be not so much a revolution as a *continuation* of molecular biology by other means (de Backer et al. 2010). The spirit is the same: the whole is still very much approached by enumerating the parts and studying their interactions, but it is now done with far more powerful experimental and computational techniques.^1^  

The recognition that the new field of systems biology, despite its prominence in the contemporary biological landscape (not to mention its ability to attract vast amounts of research funding; see Macilwain 2011), is unlikely to yield a fully-fledged system concept that can be applied to cells and organisms to better understand their holistic character has prompted disaffected biologists to look elsewhere for guidance. Some have sought refuge in what is often called *systems theory*, which is an umbrella term for a loose conglomeration of approaches stemming from a variety of sources and disciplines that emerged in the postwar period, including cybernetics, information theory, control theory, systems engineering, general systems theory, dynamical systems theory, network topology analysis, agent-based modelling, and complexity science (e.g., Barabási and Oltvai 2004; Mesarovic et al. 2004; Kaneko 2006; Dubitzky et al. 2011; Saetzler et al. 2011).

Although there is potentially much that can be learned from the application of ideas from these areas to biology, these theoretical approaches are generally perceived to be too abstract and too mathematical to be accessible—and of practical use—to experimentalists at the bench. It is not clear, then, that they can ultimately succeed (at least not by themselves) in providing what many biologists feel they most need in the present moment: a clear, workable, and straightforward understanding of what a biological system is that shows us how twentieth-century molecular biology missed the forest for the trees, and explains why it is that organisms cannot be fully understood from the ground up.

What I want to do here is offer a potential way out of this predicament by drawing inspiration from the history of systems thinking in biology—a history that has remained largely unexplored, even by most professional historians. In the early decades of the twentieth century, especially during the interwar period, a number of theoretically-inclined biologists, known collectively as the *organicists*, engaged in intense discussions about the conceptual foundations of biology in ways that resonate strongly with the sorts of questions that biologists today, almost a hundred years later, are increasingly addressing (Nicholson 2014a).^2^  

This paper rescues from the dustbin of history one of these neglected organicists, namely the Austrian-American *Paul Weiss*. Weiss was not just a refreshingly original systems thinker; he was also an accomplished experimentalist. Although he never formally presented a comprehensive systems theory of the organism, we can retrospectively reconstruct it by carefully examining his writings in a way that yields a system conception that is intuitive, non-mathematical, and is specifically tailored to the needs of current biology. This conception is markedly different from the various systems-theoretical proposals mentioned above in that it articulates the idea of a system philosophically, as opposed to mathematically, by drawing on (Weiss’s) first-hand acquaintance with cells, tissues, and organisms as experimental subjects. Instead of *projecting* onto biology a preconceived notion of what a system is supposed to be that is initially formulated elsewhere (such as in engineering or communication contexts), Weiss shows us how we can *empirically derive* a system concept of the cell and of the organism by appropriately interpreting experimental data. Although aspects of Weiss’s systems thinking have been described before (e.g., Haraway 1976: chap. 5; Drack et al. 2007; Drack 2013; Rosslenbroich 2011, 2023; Talbott 2025), my aim is to bring together his philosophical ideas about systems in a clear, digestible way so that they can be put to effective use in ongoing biological discussions.

The plan for what lies ahead is as follows. As most readers are unlikely to be familiar with Weiss, I will begin by outlining his intellectual biography and reviewing his most significant scientific contributions. Then, my task shall be to show how his philosophical insights—gathered from a lifetime of experimental work, and scattered throughout his writings—allow us to formulate a systems view capable of overcoming the major obstacles in our understanding of organisms that the molecular revolution has left us in its wake. In keeping with philosophical conventions, I will designate these obstacles using four ‘-isms’, namely ‘reductionism’, ‘genocentrism’, ‘determinism’, and ‘mechanicism’, and examine each of them in turn.

## Paul Weiss (1898–1989): Biographical Sketch

Paul Alfred Weiss was a distinguished biologist who did pioneering research in the fields of morphogenesis, differentiation, regeneration, and neurobiology over a period of more than fifty years. The precision of his observations, coupled with the elegance of his experimental designs, significantly shaped these disciplines, and also left a lasting mark on certain areas of medicine.^3^  

Weiss was born in Vienna in 1898. He grew up in a well-to-do Jewish family and developed lifelong interests in philosophy, poetry, and music (he was a classically-trained violinist). While still a teenager, he served in the Austrian army as an officer in the artillery during the First World War. In 1918 he enrolled to study mechanical engineering at the Technische Hochschule (now Vienna University of Technology), but soon shifted his studies to experimental biology after taking a course on cytology that exposed him to the work of Theodor Boveri, E. B. Wilson, and Edwin Conklin. He conducted his doctoral research under the supervision of Hans Przibram, director of the Vienna Institute for Experimental Biology (fondly known as the Prater Vivarium), completing his PhD in 1922.^4^ His thesis (published as Weiss 1925, and in English translation as Weiss 1959a) explained the response of butterflies to light and gravity in direct opposition to the prevailing views of animal behaviour at the time, exemplified by Jacques Loeb’s theory of tropisms (Loeb 1918), which regarded animals as machines and reduced their movements to automated physicochemical reflexes triggered by external stimuli.^5^  

Although Przibram appointed Weiss Assistant Director (‘Curator’) of the Vivarium following the departure of Paul Kammerer in 1924, Weiss’s *habilitation* was denied by the Austrian Ministry of Culture and Education, which prevented him from lecturing at the University of Vienna and spoiled his chances of a scientific career in Austria.^6^ In 1927 he obtained a fellowship from the Rockefeller Foundation to study cellular differentiation, regeneration, and cell growth in tissue culture at various European institutions, including the Oceanographic Institute in Monaco, the Sorbonne in Paris, and the Kaiser-Wilhelm-Institute for Biology in Berlin. He also spent six months at the Stazione Zoologica in Naples.

**Fig. 1:**
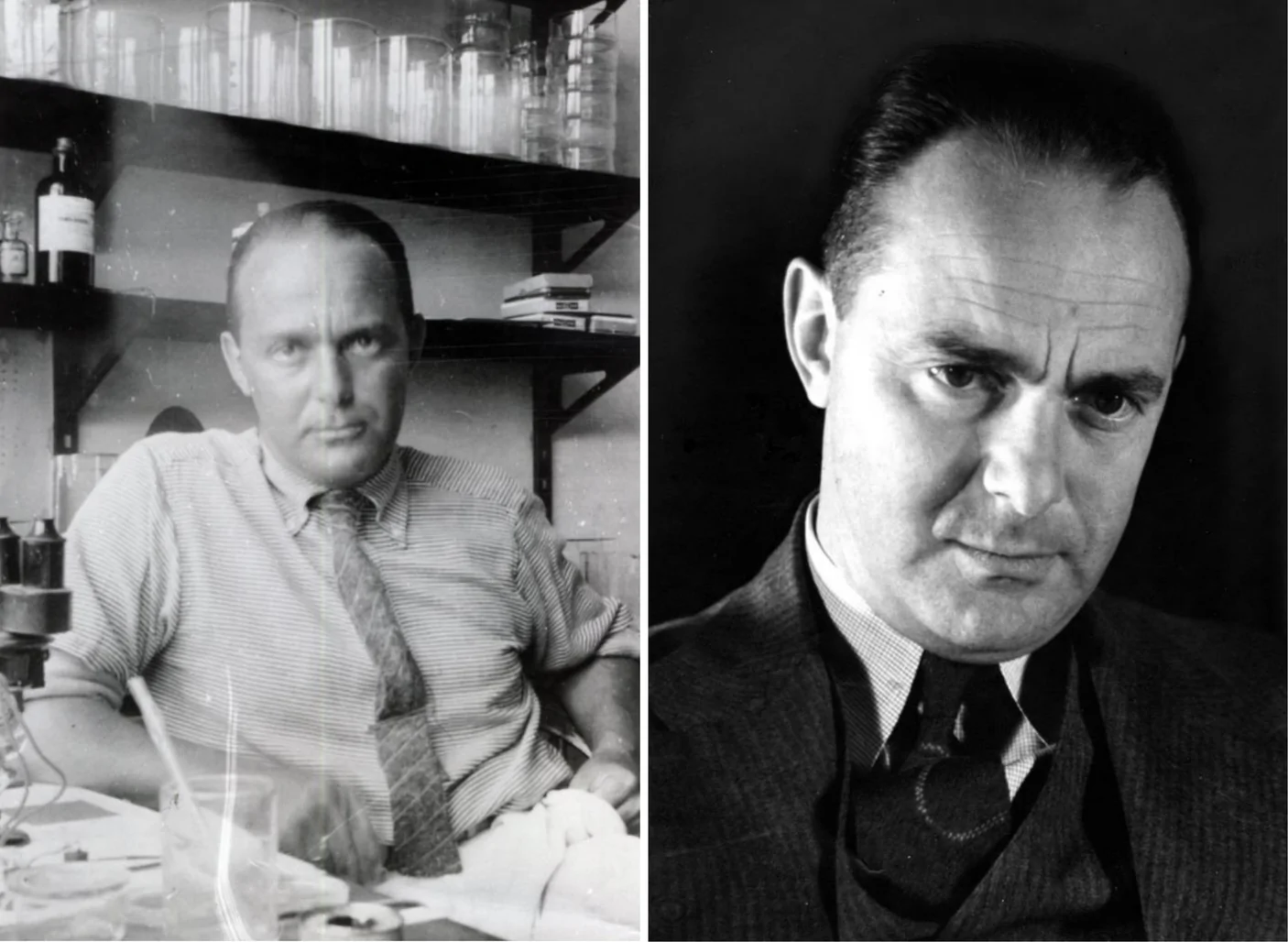
Portraits of Paul Weiss during his tenure at the University of Chicago. (Reproduced with permission of the Historical Archives of the Stazione Zoologica Anton Dohrn [**left image**] and the Special Collections of the University of Chicago Library [**right image**])

After a position at the University of Frankfurt failed to materialize in 1930, Weiss obtained a Sterling Fellowship at Yale University the following year to continue his research on tissue culture in the laboratory of Ross Harrison, now applying it to the study of nerve growth. In 1933 he was offered a position at the University of Chicago, where he taught for twenty years (Fig. 1)—being promoted to Full Professor of Zoology in 1942, and becoming Director of the Biological Division’s Master Program in 1947. At Chicago he was mostly concerned with problems related to morphogenesis, including tissue organization, organ formation, and the control of growth. He also wrote an influential, 600-page textbook titled *Principles of Development* (Weiss 1939), which was reprinted thirty years later.

Having become an American citizen in 1939, Weiss spent the Second World War assisting the United States government in the development of improved surgical techniques for nerve repair, developing the first procedure for preserving peripheral nerve tissue for later surgical grafting. He returned to pure research after the war and became one of the leading figures in developmental physiology and neurobiology, publishing widely in these areas. He was elected to the National Academy of Sciences in 1947, the American Philosophical Society in 1953, and the American Academy of Arts and Sciences in 1954. His doctoral students at Chicago included the neurobiologist and future Nobel laureate Roger Sperry and the cell biologist Howard Holtzer.

In 1954 he became one of the first professors at the new Rockefeller University in New York City, where he chaired the Department of Developmental Biology and directed a laboratory specializing in cellular differentiation, the control of growth in development, and the regeneration and repair of the nervous system. Weiss remained an active faculty member at Rockefeller for the following decade, alternating his experimental work with occasional interruptions to teach elsewhere around the world. He held appointments as Visiting Professor at ten universities on both sides of the Atlantic, and briefly served as Dean of the newly established Graduate School of Biomedical Sciences at the University of Texas in Houston in the mid 1960s. He became Professor Emeritus at Rockefeller in 1964, but he continued writing and publishing for many years after that. President Carter awarded him the National Medal of Science in 1979. He was hospitalized during the last years of his life and died after a long illness in 1989, aged 91.

Curated selections of Weiss’s most significant scientific papers (out of the several hundred that he published) were reprinted in two large anthologies: *Dynamics of Development* (Weiss 1968) and *Bio-Medical Excursions* (Weiss 1971a; republished in abridged form as Weiss 1976). Some of his humanistic writings were collected in *Within the Gates of Science and Beyond* (Weiss 1971b).

In addition to his experimental work, Weiss did a great deal to promote biological science at an institutional level. He maintained close associations with many European laboratories, and he brought numerous researchers from Europe to his laboratories in Chicago and New York. After World War II he served on a Marshall Plan commission to help revive scientific activity in Europe. At home he played a leading role in establishing the Society for the Study of Development and Growth in 1939, serving as its second president in 1942. He was later instrumental in rebranding it as the Society for Developmental Biology in 1965, and in founding the journal *Developmental Biology* in 1959. In 1962 he became president of both the Harvey Society of New York and the International Society for Cell Biology.

Perhaps most influentially, as Chairman of the Division of Biology and Agriculture of the National Research Council, Weiss was responsible for the administrative restructuring of the biological sciences within the council. In 1951 he proposed a new classification, shown in Table 1, that reflected concerns with different levels of organization, as opposed to with different forms of life (e.g., zoology, botany, bacteriology) or methods of study (e.g., biophysics, biochemistry, biometry), in the hope of facilitating the theoretical integration of historically segregated specialties.^7^ Weiss’s reimagining of American biology as a series of disciplines emphasizing principles and processes common to all life was hugely influential. It has become widely adopted globally (with some modifications) in a variety of institutional, funding, publishing, and educational settings.

**Table 1:** Weiss’s reclassification of the biological sciences in 1951 for the National Research Council

**Molecular Biology**	Concerned with the elementary compounds, their interactions, transformations, and the attendant energy balance
**Cellular Biology**	Studying the behaviour of the organized cell, especially the coordination of molecular events underlying orderly structure and function
**Genetic Biology**	Covering the laws and mechanisms of heredity, that is, the continuity of generations as well as their progressive transformation in evolution
**Developmental Biology**	Examining the growth, development, repair, and reproduction of the individual organism
**Regulatory Biology**	Dealing with coordinating and integrating mechanisms, such as homeostasis and the endocrine and nervous systems
**Group and Environmental Biology**	Dealing with the relations of individuals to one another and to their environment, including ecology, psychology, and sociology

In the last analysis, though, Weiss’s most enduring contribution to biology, as the remainder of this paper will argue, is the philosophical understanding of the organism as a system that implicitly guided his research at the bench, but which he only started to explicitly articulate in print as he began to wind down his experimental program (see especially Weiss 1962, 1963, 1967b, 1969a, 1970a, 1971c, 1973, 1974, 1977, 1978). It is Weiss’s largely neglected systems view that I will attempt to piece together in what follows with the goal of showing that it still has a great deal to offer the working biologist—not to mention the philosopher of biology.^8^  

Before proceeding, it is helpful to contrast Weiss with his more famous Viennese contemporary, Ludwig von Bertalanffy, whose work, unlike Weiss’s, does get occasionally cited by systems biologists.^9^ Bertalanffy was a pioneering theoretical biologist who, after the Second World War, went on to establish general systems theory, which, as noted in Sect. 1, is one of the fields that today’s systems-theoretical biologists draw upon. Although Bertalanffy is often credited with bringing systems thinking into biology, Bertalanffy himself acknowledged Weiss’s precedence, stating that “Paul [Weiss] was one of the first to introduce the concept of ‘system’ in biology in 1924” (quoted in Koestler and Smythies 1969: 47).^10^  

In one of his last publications, Weiss wrote about meeting Bertalanffy for the first time in the early 1920s, and recounted how he understood their different lifelong attitudes toward systems thinking:

It was in those days that a sparkling Viennese student, a little more than three years my junior, approached me for a meeting—Ludwig von Bertalanffy. We met in coffeehouses and ‘milked’ each other. I soon found that his thinking and mine moved on the same wave-length—his coming from philosophical speculation, mine from logical evaluation of practical experience. And so it remained for half a century, each of us hewing his separate path according to his predilection. That is, I kept on as the empirical experimental explorer, interpreter, and integrator, for whom the ‘system’ concept remained simply a silent intellectual guide and helper in the conceptual ordering of experience, while he, more given to extrapolations and broad generalizations, and bent on encompassing the cosmos of human knowledge, made the theory itself [i.e., general systems theory] and the applicability of it to many areas of human affairs his prime concern. (Weiss 1977: 18–19)

Despite serving only as ‘a silent intellectual guide’ for Weiss, the ensuing sections will demonstrate that his systems perspective provides a powerful and surprisingly effective antidote to some of the most pervasive yet problematic presuppositions of modern biological thought.

## Systems Thinking as an Antidote to Reductionism

Weiss’s core idea is that the organism is a *system*. What is a system? We can begin to address this question by getting clear on what a system is not. Very generally, a system can be contrasted with an *aggregate*. Both can be decomposed into parts, but they differ in the extent to which their respective parts are *interdependent*. While it is possible to change or remove parts of an aggregate without altering the condition of the others, the parts of a system interact in such a way that changing one will inevitably affect others as well. In a system, no part is truly independent of all the others (Weiss 1939: 111).

Systems also differ from aggregates in the *relationship between parts and whole*. In an aggregate, the whole has the character that it does by virtue of the character of the parts that make it up, whereas in a system, the character of the parts is at least partially determined by the character of the whole. A familiar way of expressing this contrast is to assert that an aggregate is the sum of its parts, whereas a system is *more than the sum of its parts*. This well-known maxim of holistic thought can be traced all the way back to Aristotle.^11^ Unfortunately, it has been so overused that it is likely to strike the modern reader as an empty platitude.

So let us ask: when we hear that ‘the organism is *more* than the sum of its organs’, or ‘a cell is *more* than the repository of its molecular components’, or ‘brain function is *more* than the combined activity of its neurons’, what is the ‘more’ actually referring to? The term seems to imply the *addition* of something concrete and quantifiable.^12^ The question thus becomes: more of what? Volume? Mass? Energy? It is clearly none of those. What, then, is intended? Perhaps something inscrutable, dimensionless, non-physical? The history of biology is replete with vitalistic proposals of precisely this kind (e.g., *Lebenskraft*, *Bildungstrieb*, *entelechy*, *élan vital*), but these turn out on closer inspection to be little more than extravagant labels for an unknown X.

The embarrassment caused by the impotence of such pseudo-explanatory labels has tended to push biologists to the opposite extreme—throwing the baby out with the bathwater by sweepingly rejecting any suggestion that a biological whole might be anything more (or anything other) than the sum of its parts. It has resulted in the widespread adoption of *reductionism*, interpreted here as an explanatory strategy which prescribes that a whole can be fully understood by analytically decomposing it and then progressively piecing together all that can be learned from a study of its elements and their interactions (Weiss 1967b: 801). This was, of course, the guiding principle of the molecular revolution. As François Jacob famously declared, “[t]he aim of modern biology is to interpret the properties of the organism by the structure of its constituent molecules” (Jacob 1973: 9). “Complete certainty now exists”, James Watson remarked, “that the […] characteristics of living organisms […] will all be completely understood in terms of the coordinate interactions of small and large molecules” (Watson 1965: 67).^13^  

**Fig. 2:**
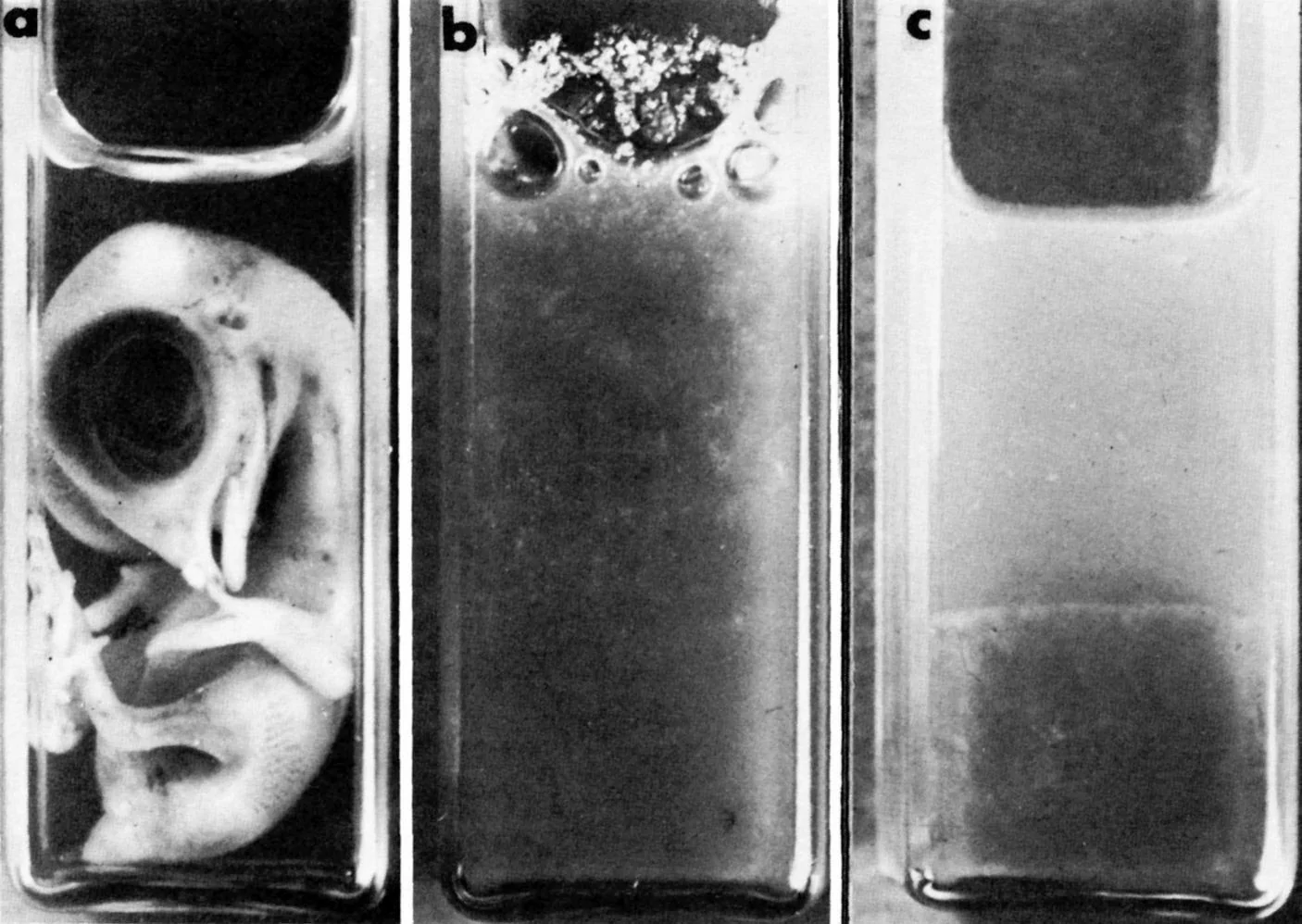
Chick embryo: (**a**) intact; (**b**) homogenized; (**c**) fractionated (after Weiss 1962)

Recognizing the system character of an organism prevents us from capitulating to the reductionistic credo of modern biology. It tells us that exhaustively characterizing the parts is not sufficient—even in principle—to practically or mentally reconstitute the whole. Why not? Of what are we depriving a system when we break it down that needs to be restored for us to arrive at an understanding of the whole?

Weiss liked to illustrate this problem in his lectures at Chicago by showing his students a six-day-old chick embryo in a balanced salt solution before (Fig. 2a) and after (Fig. 2b) it is ground up into a homogeneous suspension. As nothing is added or removed during the grinding process, the content of the vial before and after is exactly the same with regard to weight and composition. An exhaustive inventory of all the molecules, if it could be taken, would likewise reveal no change.

Getting from Fig. 2a to 2b is easy; experimental biologists do it all the time. The real question is how to go back from Fig. 2b to 2a. If we understand the change from Fig. 2a to 2b as the entropic transition from a heterogeneous to a more homogeneous distribution, we can reverse the direction by subjecting the homogenate of Fig. 2b to centrifugation. This separates the suspension into fractions according to their weight, as shown in Fig. 2c. Notice, however, that the outcome is not a step back toward the lost old order of Fig. 2a, but a step forward toward a new and artificial form of order—one that brings us no closer to understanding the whole we started off with (Weiss 1962: 8–10).

It seems preposterous to seriously entertain the notion that the chick could be re-assembled from the mush produced by our analysis of it.^14^ Yet for those beholden to the reductionist ideology of molecular biology, it is not exactly clear why this should be so. After all, does the homogenate in Fig. 2b not contain all the *genetic information* of the chick? And not just the DNA sequences, but also the enzymes needed to transcribe them into RNA, as well as the ribosomes required to synthesize the corresponding proteins? Would it not be logical, then, to suppose that the homogenate, comprising all the necessary molecules and genetic information, could in principle be reconstituted to generate an organism? (Weiss 1977: 26).

There are numerous problems with this fanciful proposition, many of which will be examined later. For now, it suffices that we realize the obvious, which is that what has been lost in moving from Fig. 2a to 2b is the system’s *organization*—from the highest level of the organism down to the level of whatever structures were small enough to have eluded the disruptive forces of the grinding process. Organs and tissues have been broken up, and so has the internal architecture of the individual cells. All the material is still there, but the specific structural order upon which the functional capacity of the organism depends has disappeared.

What is true for a chick embryo is true for a single cell, as both are systems. Viewed solely in terms of its composition, a cell is nothing more than the sum of its molecular parts. Because analytical decomposition produces basically the same result for a living cell as it does for a dead cell, and even for the homogenate of a ground up cell, a reductionist approach inevitably overlooks much of what makes the cell the kind of entity that it is. Indeed, it effaces the very basis upon which we distinguish a live cell from a dead one, a dead one from its homogenate, or a homogenate from its fractionated parts (Weiss 1963: 389).

In a memorable (yet slightly dated) analogy, Weiss remarks that characterizing the life of a cell by an inventory of its molecules is as inadequate as characterizing the life of a city by the list of names and numbers in the telephone directory (Weiss 1969a: 8). In another striking analogy drawn two decades earlier (Weiss 1949: 147), he observes that just as a museum collection of animal specimens is not life-like unless it shows them in their natural environment and ecological relations, so is a molecular catalogue obtained by analysis not representative of a cell unless it is supplemented with information about the interactions, associations, and general operating conditions that tie together the various molecular species into a durable and viable ‘community’ (we will return to this intriguing idea of a ‘molecular ecology’ in Sect. 5).^15^ What follows from these evocative illustrations is the realization that an understanding of the *operation* of a system cannot be inferred from a comprehensive description of its *composition*.

This brings us back to the holistic maxim with which we started, as we are now in a better position to understand the precise sense in which the whole is more than the sum of its parts. The ‘more’ does not refer to any ontological, measurable quantity in the system. Instead, Weiss argues, it refers to the epistemic necessity to supplement the knowledge obtained from the study of the parts by the knowledge required to appropriately describe the *collective behaviour* of the parts when these are considered as a coordinated unit. To speak of an integrated system, as opposed to a mere sum of parts (like an aggregate), involves no physical—or, for that matter, metaphysical—addition to the whole. Weiss makes this point most eloquently in an essay with the wonderfully provocative title ‘1 + 1 ≠ 2 (When One Plus One Does Not Equal Two)’ (Weiss 1967b). I will have more to say about this holistic maxim later in the paper.

The enthusiastic embrace of reductionism in the biological study of organisms has meant that their organization, and even their *behaviour*, have received far less attention than their material constitution. But understanding the organism as a system allows us to appreciate their fundamental importance. In fact, it was primarily the study of animal behaviour that prompted Weiss to first propose his systems view of the organism in 1922. His doctoral thesis, published with the telling title ‘Animal Behaviour as System Reaction’ (Weiss 1925, 1959a), argues that organisms respond as *unitary entities* to external cues, exhibiting far more plasticity (this is the term Weiss uses) in how they accomplish their goals in variable conditions than would be expected if their behaviour resulted from a rigid concatenation of stereotypical reflex reactions of their parts, as Loeb and others held at the time. Organisms, as Weiss (1959a [1925]: 3) puts it, “are determined only for a certain general mode of motion; in detail, however, their motions are able to adapt themselves to the circumstances prevailing at each movement, which of course, would be wholly impossible in the case of a strictly mechanical stereotypism [as proposed by Loeb]”.

Weiss also notices that organisms are able to compensate against external perturbations—they maintain their unitary identity despite incurring modifications to their parts. This leads him to offer a definition of ‘system’ (the first of many, as we will see) in terms that emphasize *self-regulation*: “A system may therefore be defined as any complex which tends to retain its constancy as a whole relative to the outer world during and not withstanding [sic] the changes taking place in its parts” (ibid.: 8).^16^  

The self-regulating behaviour of systems is something that Weiss continued to stress throughout his career. For example, in his 1939 textbook on development, he remarked that a developing embryo “act[s] in such a manner as to preserve the state of the *system as a whole* throughout the fluctuations to which its constituent parts are continually subject”, adding by way of contrast that “[w]hile a mere aggregate would fall apart with no sign of inner coherence, a system [such as an embryo] obstinately tends to retain, and return to, its internal configuration” (Weiss 1939: 112). Many decades later, Weiss defined ‘system’ along similar lines as “a complex unit in space and time so constituted that its component subunits, by ‘systematic’ cooperation, preserve its integral configuration of structure and behavior and tend to restore it after non-destructive disturbances” (Weiss 1971c: 14).

It should be clear how these distinctive features of systems militate against the prescriptive use of reductionism as a sufficient explanatory strategy in biology. If an organism reacts in a unitary way to external cues, then the principles that govern its behaviour need to be investigated *at the level of the whole*. And if the behaviour of the parts is not solely determined by their own properties, but is instead shaped by the system to which they belong, then what the parts do cannot be explained without attending to the whole. The conditions of the whole must be ascertained in order to understand the coordinated behaviour of the parts; it is not an understanding that can be arrived at reductionistically by simply describing the parts and their interactions in increasing levels of complexity.

## Systems Thinking as an Antidote to Genocentrism

Another core doctrine of modern biology is *genocentrism*—the belief that genes are central to all organismic processes: from development to physiology to behaviour (and ultimately to evolution). Genocentrism can be regarded as a particular form of reductionism, inasmuch as it reduces biological complexity to the action of genes, but it is sufficiently distinct to merit independent consideration. It is, of course, a foundational principle of molecular biology, enshrined in famous tenets like the ‘Central Dogma’ (Crick 1958) and the ‘genetic program’ (Jacob and Monod 1961), though the basic idea predates the rise of molecular biology (Muller 1929; Schrödinger 1944), and arguably even the advent of genetics (Weismann 1893).^17^ The aim of this section is to explore how the perspective afforded by the system concept allows us to identify and circumvent the problems with a genocentric view of the organism.

How does a system come into being? Unlike an aggregate, it does not arise from the assembly of pre-existing parts. The reason is that in a system the network of interactions between the parts is causally responsible for the generation of each part. Consider, as the simplest possible example, a triplet of interdependent processes—A, B, and C—each of which provides some of the conditions required for the production of the two others. This triplet cannot be assembled by simply adding A + B + C in a stepwise manner, given that no two members of the set—(A + B) or (B + C) or (C + A)—can exist or be maintained without the presence and contributions of the third member—C, A, or B, respectively. The *coexistence and cooperation of all three* is indispensable for the existence and operation of any one of them (Weiss 1969a: 17–18, 1973: 76). This key feature of the organization of living beings has received a great deal of attention in the theoretical biology literature in recent decades, where it is usually described as a form of *closure* (Varela 1979; Rosen 1991; Kauffman 2000; Moreno and Mossio 2015).^18^  

**Fig. 3:**
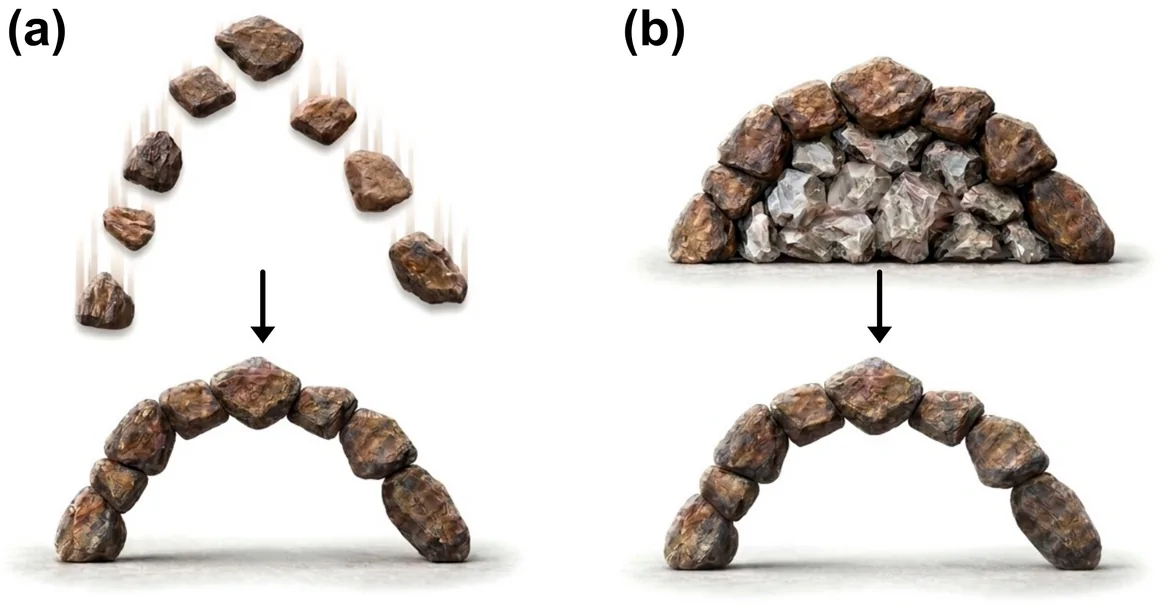
Two hypotheses for the origin of an arch: (**a**) the stones fell spontaneously into place to create an arch; (**b**) the arch arose above a scaffolding of supporting stones that is no longer present (based on Cairns-Smith 1985b; after an analogy described in Weiss 1959a [1925] and 1969a)

We can illustrate this interdependence of parts with the analogy of an arch of stones (Weiss 1959a [1925]: 11, 1969a: 18). One cannot build an arch by adding the loose stones that make it up one at a time, as the position and stability of each stone is dependent on the structural integrity of the whole. As a self-supporting structure, the arch *can only exist in its entirety or not at all*. Though static, the arch shares this property of systems with organisms. For this reason, Weiss’s architectural analogy has been invoked in discussions about the origins of life (e.g., Cairns-Smith 1985a), as it provides a helpful way to think about the origin of systems. It seems very unlikely that an arch could spontaneously come into existence by all of its constituent stones fortuitously falling into place (Fig. 3a). The reliable way to build an arch is to position a temporary scaffolding of supporting stones underneath until the structure of the arch becomes stabilized, at which point the scaffolding is no longer needed and can be removed (Fig. 3b). Similarly, it seems reasonable to suppose that the organization of the first cells did not emerge spontaneously, but rather arose incrementally by means of complex biochemical scaffolding that was later lost in evolution.

As far as present-day cells are concerned, their systemic organization explains why they only arise from pre-existing cells. Without the existing organization to serve as a template, the extraordinarily complex web of interdependent processes that make up a cell cannot be recreated *de novo*. The cross-generational continuity of organization is an essential aspect of biological reproduction, even if it is not always appreciated (see Griesemer 2000). Weiss (1940: 46) proclaims that Rudolf Virchow’s famous tenet of cell theory—*omnis cellula e cellula* (‘all cells come from cells’)—ought to be supplemented with its organicist counterpart: *omnis organisatio ex organisatione*. Only systems beget systems.

Let us now see how genes fit into this picture. Genes are often described by their ability to *do* things. They are said to be ‘self-replicating’, to ‘make’ proteins, to ‘switch on’ molecular pathways, and so on. But it becomes clear that these expressions are misleading when we recall that, chemically speaking, DNA is one of the most inert and nonreactive molecules in the cell (which helps protect the genetic message stored in its nucleotide sequence from thermal fluctuations). Alone, DNA does nothing. To be functional, it needs to be embedded in an organized cell. In other words, DNA does not function by imparting order on an orderless cellular milieu; it functions only because its cellular milieu is *already ordered* (Weiss 1969a: 37). New DNA strands are replicated by a pre-existing molecular apparatus that uses the old DNA strands as templates. Transcription and translation are likewise heavily scaffolded processes, requiring the coordinated involvement of many other molecules in the cell, especially proteins. Paradoxically, this means that gene expression relies on its own outcome—the synthesized proteins—to take place. Yet only when we close this circle of interdependent processes to form an integrated system are we justified in using words like ‘*self*-producing’ and ‘*self*-maintaining’ to describe it (ibid.: 19).

Other problems with genocentrism derive from misunderstandings of the methodology of genetics. Genetic analysis proceeds by *comparing differences* among members of a population with respect to observable characters or traits. We should not forget that the very idea of a gene was introduced in the early twentieth century to serve as a tool of analysis. At first, genes were nothing more than intervening variables that helped classical geneticists track the patterns of inheritance they observed in their crossbreeding experiments. Only decades later, with the rise of molecular biology, did the gene concept come to refer to specific DNA sequences, thereby acquiring a new meaning in cellular and developmental contexts. But genes have never stopped serving as markers of phenotypic differences. They are still frequently understood in this way, for instance, in the context of clinical genetic testing.

Consider ‘gene for’ locutions. When we speak of ‘genes for blue eyes’ or ‘genes for brown eyes’, we are referring to whatever is transmitted from one generation to the next that reliably correlates with an observable difference in eye colour between otherwise similar individuals. We are not necessarily referring to specific sequences of DNA responsible for the formation of blue eyes or brown eyes. In fact, at the molecular level, *there are no genes for blue eyes*. Blue eye colour is what you get when you lack the genetic resources to produce enough melanin to make brown eyes. So we can use genetic analysis to account for why an individual has blue eyes *as opposed to* brown eyes, but not to explain why an individual *has* eyes. The formation of the eye is a complex developmental process involving the confluence of many intersecting causal factors, only some of which have their basis in DNA. The same can be said for any other organ in the body. One cannot arrive at an explanation of how an organ forms by describing all the genes that can modify its attributes. It follows that an organism’s development cannot be fully explained in terms of the genes that are known to play a role in it (Weiss 1969a: 34–36).^19^

The explanatory overreach of genocentrism in these and other cases reflects the peculiar way that experimental biologists handle *causes* (see Weiss 1974, 1978). The famed biochemist Albert Szent-Györgyi once jokingly defined a vitamin as ‘a substance that makes you ill if you don’t eat it’. In a similar vein, genetic causation is typically ascertained by the negative effect that the *absence* of a gene has on a physiological process. If the knockout or modification of a gene is found to *disrupt* X, then the gene is *identified* with X, and is even said to have *caused* X. BRCA1, for example, is a well-known ‘gene for breast cancer’. But the protein it expresses does not produce breast cancer. On the contrary, its normal function (which is to protect the integrity of the genome) helps *prevent* cancer: it is a tumour *suppressor* gene. It is the *impairment* of its normal function that is statistically correlated with the onset of breast cancer—it increases its likelihood, though it is neither necessary nor sufficient.^20^ Yet even in simpler, more clear-cut cases, the most that one can establish between a gene and a given phenotypic outcome is necessary participation, never exclusive responsibility. It is evidently not appropriate to infer the latter when only the former is warranted.^21^ A gene can therefore be understood in a *negative* sense as something *without* which some trait that you expect *fails* to develop, resulting in some *other* outcome instead. But, Weiss (1970a: 129) adds, “[t]o turn the sum of such negative statements around and fashion from them a positive doctrine of plenipotency (of […] genes) seems to me a reprehensible somersault of logic”.

Nevertheless, this ‘doctrine of plenipotency of genes’ (or genocentrism, as we are calling it here) is exactly what one finds in modern biology. DNA has been elevated to the status of a ‘master molecule’; it sits at the heart of the cell as an autocratic ruler directing all of life’s processes. Indeed, it has become almost synonymous with life itself. When in 2010 Craig Venter succeeded in synthesizing a bacterial genome and inserting it into a host cell previously stripped of its own genome, this feat was widely heralded in the scientific and mainstream press as ‘the artificial creation of life’ (see, e.g., Rusk 2010; Elowitz and Lim 2010), despite the fact that the function of the synthetic DNA relied on the pre-existing organization of the host cell. To presume that what makes a cell alive is its genome, as opposed to its self-maintaining organization, is to neglect the fact that life is a property of *systems*, not of molecules. The systems perspective reminds us that “the basic criterion of cell life lies in the intricate web of interactions and interdependencies among *all* of its component activities” (Weiss 1963: 391). For Weiss, “it is precisely this indissoluble interdependence among its various component operations which […] discourages prospects for a ‘synthetic cell’” (Weiss 1962: 3).

Unfortunately, instead of candidly acknowledging the limits of what genes can do (as evidenced, say, by what happens when DNA is removed from its cellular milieu), the general preference has been to take recourse to pretentious anthropomorphic terminology to fill the gaps in our understanding of complex phenomena—in the hope that genes will eventually be found to be responsible for them. Many biologists feel no compunction about endowing genes (or the proteins they express) with whatever faculties observed at the level of the whole need to be explained. It is striking, though regrettably not at all unusual, to see molecules being vested with powers to ‘control’, ‘dictate’, ‘command’, ‘orchestrate’, and so on.

A provocative (though, I think, accurate) label to describe this way of thinking is *molecular animism*. Whereas the animistic theories of the past endowed living matter with souls or vital principles, which acted on the body from the *top down*, today’s molecular animism bestows this agency upon genes and molecules, which exercise their authority from the *bottom up*. Molecular animism is everywhere. Developmental biologists routinely express it when they speak of ‘master control genes’ (e.g., Gehring 1998), and it is a common trope in popular science, such as in Richard Dawkins’ bestseller *The Selfish Gene* (1976).

Weiss began criticizing appeals to what I am calling ‘molecular animism’ in the late 1940s and early 1950s (Weiss 1949: 135, 1953b: 173–175), and he continued doing so later on in his more theoretical writings (e.g., Weiss 1969a: 36–37, 1973: 10, 19, 69).^22^ Here is how Weiss phrased his criticism in 1963:

The common habit of personifying compounds by calling them ‘regulators’, ‘integrators’, ‘organizers’, etc., and crediting them verbally with the ‘regulatory, integrative and organizing’ effects which one observes but cannot explain analytically, either intends to endow chemicals with spiritual powers up and above their ordinary properties, or else is wholly meaningless. To state it bluntly, it would be rather a reversion to the prescientific age if on observing, for instance, the spinning of a whirl of fluid, one were to invoke a special compound as ‘spinner’. (Weiss 1963: 395)

The reason this language is so misleading is that it drastically overextends what can be legitimately inferred from genetic and molecular studies. And the problem is as acute today as it was in Weiss’s time. Take gene knockout experiments, which we mentioned briefly in our discussion of causation. Although such experiments can only really show that the expression of a gene is *necessary* for the occurrence of a given process, all too frequently they are marshalled in support of the much stronger conclusion that the gene in question is the ‘regulator’ or ‘controller’ of that process (see Davies 2009 for a recent critique).

Molecular animism encourages us to imagine the cell as an inert playground for a few almighty masterminding molecules. But the logical coherence of this picture falls apart when we bear in mind that, as a system of interdependent processes, the cell’s organization leaves no room for any particular molecule to exert monopolistic control over all the others. Attributing *collective* properties of the parts that make up a system—such as coordination, organization, and agency—to *individual* parts in that system is a category mistake, albeit a surprisingly common one. This mistake arises from the failure to recognize a point we made at the end of our consideration of reductionism in Sect. 3, namely that what a part of a system does is shaped by the influence exerted upon it by the system to which it belongs.

Weiss makes this clear with respect to the relation between the cell and its constituent molecules in a 72-page paper titled ‘From Cell to Molecule’ (Weiss 1962). In it, he shows how a careful examination of examples that purport to demonstrate the *molecular control of cellular activity* invariably reveals that the ‘controlling’ molecules have acquired their ‘powers of control’ by virtue of their membership in the organization of the cell, and are thus themselves subject to ‘cellular control’. In other words, what such examples actually illustrate on closer inspection is the *cellular control of molecular activity*.

What we have just said about the relation of genes to the cell is equally relevant to understanding the relation of genes to the developing embryo. Here, too, the tendency to project onto genes animistic powers to ‘initiate’, ‘dictate’, or ‘control’ development betrays a lack of understanding of the systemic character of the process. Genes are certainly necessary for development, but they do not singularly trigger it or determine its outcome. In this context, Weiss (1971c: 34) advises us to be mindful of the difference between asserting that a phenomenon is ‘gene-related’ and asserting that it is ‘gene-determined’.

The belief that an organism’s phenotype is specified in advance by its genetic makeup—an idea that harkens back to the old doctrine of *preformation*—is a particularly fraught manifestation of genocentrism.^23^ Although the distinction between ‘genotype’ and ‘phenotype’ was originally introduced to emphasize the contrast between genetic and morphological (or behavioural) descriptions of the organism, in more recent times the two concepts have taken on preformationist overtones. It is as if, Weiss (1970a: 96) observes, the genotype corresponded to an ideal Platonic type, and the phenotype reflected its imperfect or corrupted realization resulting from the interference of the environment. All that the environment does is botch the execution of a pre-existing genetic design. Environmental influences on development are treated as “a sort of contamination, which if removed would then reveal […] the pure genetic product in its pristine unblemished glory” (ibid.: 144).

Although differences between traits can be ascribed to differences between genomes, and the lack of a particular trait can be ascribed to a mutation in a particular gene, in a *positive* sense, no trait is a pure ‘gene product’. Genes always exert their influence in tandem with the environment that engulfs them. It makes no sense to pit genes against the environment by asking whether a given trait is genetic or environmental—much less by trying to assign numerical values to the respective developmental contributions of genes and the environment. Such efforts, Weiss (ibid.) writes, are “about as unscientific as if one were to express the volume of a gas, which is proportional to the ratio of temperature over pressure, by crediting a given number of cubic centimeters to temperature, and what is left, to pressure”.

**Fig. 4:**
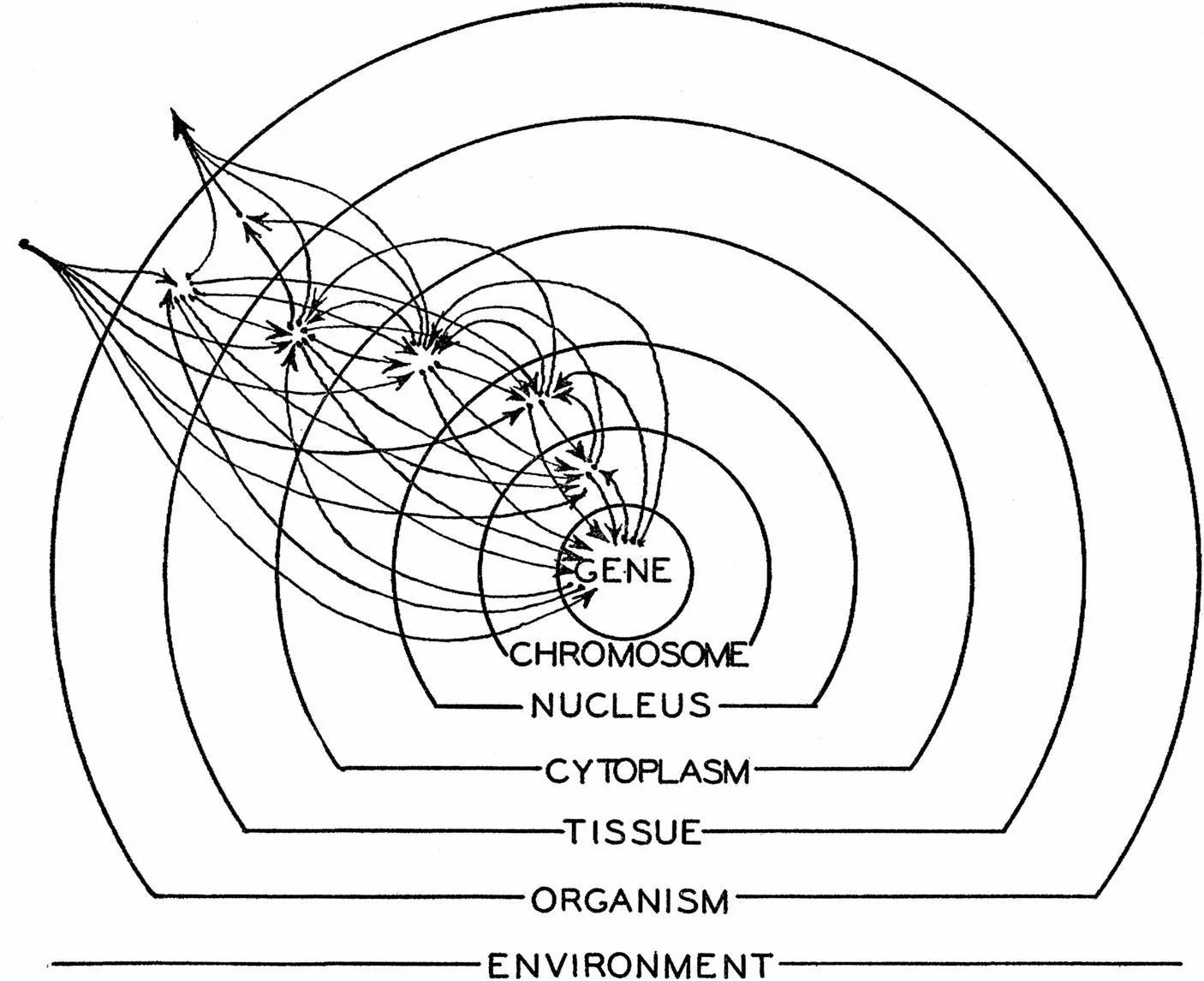
Diagram representing the wide range of potential interactions in the hierarchical organization of an organism or a developmental system (after Weiss 1950)

More fundamentally, when we ascribe a trait to *either* genes *or* the environment, we are imposing an artificial dualism which implies that all developmental resources that are not genetic are thereby environmental. This leaves us with a hopelessly vague concept of the environment, and it overlooks the fact that genes constitute only one level in the *hierarchical organization* of a developmental system. Figure 4 shows a diagram that first appeared in Weiss 1950—and which Weiss reused in many of his later publications (e.g., Weiss 1959b, 1969a, 1971c, 1973, 1978)—that schematically represents the developmental system as a hierarchy of concentric shells nested in descending orders of magnitude. Each shell affects, and is in turn affected by, its inner and outer neighbouring shells. Genes are embedded in the chromosomes, the chromosomes are contained in the nucleus, the nucleus is surrounded by the cytoplasm, the cells are ensconced in tissue matrices, which are themselves bathed by the internal milieu of bodily fluids (blood, lymph, etc.), and the organism as a whole is subject to the external environment.

Notice that for each shell, the ones outside of it make up the ‘environment’ in which it is enveloped. And because all shells are porous (i.e., none is completely isolated), no part of the hierarchy can be singled out and treated as if it were independent of the rest—*let alone in control of them*. Each part can only be properly described in relation to the environment with which it dynamically and reciprocally interacts. Developmental biologists should therefore investigate the entire web of possible interactions that are symbolized by the arrows shown in Fig. 4.^24^ In a remarkable anticipation of what has come to be known as *developmental systems theory* (Oyama 1985; Griffiths and Gray 1994; Oyama et al. 2001), Weiss (1971c: 39) asserts that “the developmental system is a ‘system’, in which the genes do not ‘act’ as independent autonomous dictators, but with which they simply ‘interact’ as cooperative parts” with the rest of the elements in the system.

As a final point before we wrap up our discussion of genocentrism, it is worth emphasizing that Weiss was also one of the earliest and most perceptive critics of the adoption of the concept of information in biology. Writing in 1971—at the height of the molecular revolution—Weiss complains that the genocentric belief that genes are the source of organismic order is an “illusion created by the thoughtless and careless use of the term ‘*information*’ rampant in current literature and as misleading as a mirage in the desert” (ibid.: 35). The problem, he argues, has arisen due to “a naïve misapplication of the principles of valid information theory” (ibid.: 36), which, he adds elsewhere, “must not be blamed when its name is being taken in vain” (Weiss 1973: 62). Weiss notices that, just as with the metaphorical recourse to anthropomorphic language discussed earlier, information-talk is often invoked in biology when there is an *absence* of understanding about how a phenomenon comes about. In such cases, the appeal to “the magic cure-all of ‘information’ verbiage” is, ironically, just “a pretentious cloak for ‘lack of information’” (ibid.).

The trouble with thinking that genetic information is responsible for development (or anything else) is that information itself is not agential; it must be read, interpreted, and translated into reality by external agents—that is, by *systems*. Genetic information does not *make* proteins, let alone organisms. “Machines and houses are built *from* blueprints (‘information’ charts)”, Weiss quips, “but not *of* blueprints” (Weiss 1970b: 161). Information is likely indispensable, yes, but it cannot be the whole story. To fully account for the development of an organism, “genetics must call on *systems dynamics* for supplementation” (Weiss 1969a: 39). Even at the level of the individual cell, Weiss remarks, “the worm’s eye view of ‘*molecular* biology’”, which approaches it from the bottom up, must be “supplemented by the bird’s eye view of […] ‘*cellular* biology”, which considers it from the top down (Weiss 1961: 5).^25^  

## Systems Thinking as an Antidote to Determinism

Returning once more to the relationship between parts and whole, a distinctive feature of systems is that the *dynamics* of the parts are fundamentally different from the dynamics of the collective they make up. While the parts of a system typically enjoy considerable spontaneity and freedom, the collective they form acts to *constrain* those freedoms so that their dynamics converge on regular, non-random outcomes. The orderly behaviour of a system is established from the top down, rather than from the bottom up. Yet what a system does is not the summative result of all of its parts interacting with one another in an automated fashion by following a fixed concatenated sequence of operations. As Weiss explains,

the systems concept is the embodiment of the experience that there are patterned processes which owe their typical configuration not to a prearranged, absolutely stereotyped, mosaic of single-tracked component performances, but on the contrary, to the fact that the component activities have many degrees of freedom, but submit to the ordering restraints exerted upon them by the integral activity of the ‘whole’ in its patterned systems dynamics. (Weiss 1969a: 9)

A system acts as a unit by subordinating the activities of its parts so that, instead of being independent and unrelated, they are restricted and directed toward the coordinated execution of tasks performed by the whole. It is the orderliness of these restraints which imposes upon the collective the overall regularity of form, function, and behaviour that sets a system apart from an aggregate of randomly interacting elements (Weiss 1960: 182–183).

A living cell perfectly exemplifies this. Although there are countless ways in which the millions of molecules it contains can potentially interact with one another, only an infinitesimal fraction of this vast range of possibilities is actually realized in the lifetime of a cell—and it is the dynamics of the collective that imposes such tight constraints on what can happen inside it. This gets at what Weiss means by the ‘cellular control of molecular activity’ (Weiss 1962: 1), which we mentioned in our discussion of genocentrism. In fact, it was his frustration with the genocentric view of cellular order that prompted him to propose a radically different way of conceptualizing the internal dynamics of the cell, which he called *molecular ecology* (see Weiss 1947, 1949, 1950).

The concept of molecular ecology draws on the ways organisms interact in an ecosystem to shed light on how molecules interact in a cell. Just as in an ecosystem, the ‘community’ that constitutes a cell is highly heterogeneous, as it is made up of many different kinds (or ‘species’) of molecules that vary enormously in number, size, composition, behaviour, and stability. The individuals in the community are not self-sufficient, but rather depend to varying extents on their associations with other members of their own species, as well as on ‘symbiotic’ relations with members of other molecular species—relations that can be cooperative or competitive, transient or permanent, ‘obligate’ or ‘facultative’, and ‘mutualistic’, ‘commensalistic’, or ‘parasitic’. Individuals that rely on one another tend to cluster together in the same locations, and as a result different molecular groupings naturally segregate (i.e., ‘self-organize’) into disparate regions (or ‘niches’) characterized by particular physicochemical conditions, which are themselves ‘constructed’ in part by the activities and products of the individuals that occupy them (cf. Odling-Smee et al. 2003). The patterns of interaction, association, and distribution among the different factions of the community are constrained and sustained dynamically by the ‘ecological conditions’ that the various clusters of molecules create for one another within the bounded physical space of the cell.

Molecular ecology transcends the biochemical study of particular molecules in the same way that ecology transcends the ethological study of individual organisms. One of its principal theoretical virtues is that it gives expression to the fact that a cell is neither a “random scramble of molecules, nor, at the other extreme, a rigid stereotyped composite of microstructures, but something in between; part fluid, part consolidated” (Weiss 1963: 394). The molecules that make up a cell do not exhibit the unlimited degrees of freedom of interaction they would otherwise enjoy in a dispersed state of thermal agitation (such as in a test tube solution), but neither is their location and behaviour strictly imposed from the bottom up by a ‘master molecule’ in control of cellular operations. Accordingly, molecular ecology allows us to distinguish an organized cell from its homogenate in a way that an exclusive focus on material composition or genetic information does not (recall the contrast between Fig. 2a and 2b). It also tells us that the order of the cell does not specify the precise structure and position of all molecules, but “only the general statistical norm of the[ir] proportions, arrangement, and distribution” (Weiss 1949: 149). What this means is that we “must seek the source of this order in the *population dynamics* of the cellular constituents” (Weiss 1962: 7).^26^

Weiss mentions at one point that molecular ecology is “essentially another version of the systems concept” (Weiss 1971c: 41), and it is easy to see why. Its characterization of the cell instantiates the general considerations of systems with which we began this section, when we indicated that the dynamics of the parts of a system are fundamentally different from the dynamics of the collective they make up. One of the most significant respects in which a cell—again, just like an ecosystem—differs dynamically from its parts is that it remains recognizably similar to itself over time despite the fact that its constituents are concurrently undergoing incessant reshuffling and *turnover* (metabolic in the case of a cell, generational in the case of an ecosystem). A cell retains its identity as a system while everything inside of it changes: small molecules come and go, macromolecules are synthesized and degraded, the cytoskeleton assembles and disassembles, cargo is transported and processed, organelles divide and merge, and so on. The sheer extent and pervasiveness of change is so overwhelming that it is safe to say that at no point in the lifetime of a cell (much less in comparable stages of different cells) do the same constellations of molecular constituents ever precisely reoccur (Weiss 1962: 6).^27^  

“[T]he basic characteristic of a system”, Weiss notes, “is its essential *invariance* beyond the much more variant flux and fluctuations of its elements” (Weiss 1969a: 12). He codifies this as a general principle of “order in the *gross* with freedom of excursion in the *small*” (Weiss 1955a: 297). This contrast between parts and whole is a crucial feature of the dynamics of systems. We can express it mathematically by considering the following thought experiment. Suppose we focused on part *a* of a system *S* and measured all the fluctuations from the mean for a range of physical and chemical parameters over a given period of time, designating the cumulative record of these deviations as the total *variance* of *a*, or *v*_*a*_. Suppose we then carried out the same procedure for all the other parts that make up *S*—*b*, *c*, *d*, …, *n*—thereby identifying their respective variances *v*_*b*_, *v*_*c*_, *v*_*d*_, …, *v*_*n*_. Lastly, suppose we measured the same parameters of *S* over the same time period, determining its own variance *V*_*S*_. What we would invariably find, if we studied any system in this way, is that the sum of the individual variances of the parts is *far greater* than the total variance of the whole—or, written in a formula:

*V*_*S*_ ≪ ∑ (*v*_*a*_ + *v*_*b*_ + *v*_*c*_ + *v*_*d*_ + … + *v*_*n*_)

This is the only mathematical expression we find in Weiss’s theoretical writings about systems, but he uses it often (e.g., Weiss 1963, 1969a, 1971c, 1973, 1977, 1978), sometimes as a way of illustrating how one might go about empirically identifying systems in nature. Still, its importance for us lies in that it captures a key aspect of all systems that biologists are confronted with, not just cells. Indeed, it holds for systems *at any level *of the biological hierarchy. Just as the molecules behave far more freely and variably than the cell they make up, so do the cells that compose a tissue, the tissues that form an organ, the organs that constitute an organism, and the organisms that comprise a population.^28^ At every one of these levels, a system retains its ordered state by coordinating the activities of its parts, restraining their degrees of freedom in a way that helps maintain its stability, just as its own degrees of freedom are in turn restrained by the pattern of activities of the higher system of which it is a part. Each level exhibits more regularity and invariance than the one below it, but less so than the one above it.

These considerations of systems dynamics have a direct bearing on the adequacy of *determinism*, which is another common presupposition of modern biological thought. Prima facie, an organism is such an extraordinarily complex entity, exhibiting so much order and regularity at the macroscopic level, that it seems reasonable to suppose that the microscopic processes upon which its viability depends must unfold in a very precise manner following an intricate chain of tightly controlled events, with each of them being a necessary precondition for the occurrence of the next. This is, in fact, what we find when we examine the workings of other highly organized macroscopic entities, such as machines. What makes the activity of a machine so regular and predictable is that its parts interact in an extremely specific way; the mechanisms that make it up have been designed to always execute the same exact predefined cycle of operations. In other words, the *macrodeterminacy* of a machine’s function is simply a reflection of the *microdeterminacy* of its component mechanisms.^29^  

Crucially, however, macrodeterminacy need not be grounded in microdeterminacy. A process can be fully determinable at the macroscopic level (in the sense that, if we understand its nature and know its antecedent conditions, we can predict its outcome—the confidence in our prediction resting on countless past observations) *even if* the individual microscopic events that constitute it are unpredictable (because, being nonrecurrent and unique, they have no exact precedent in our experience). Physicists have known this since Ludwig Boltzmann established the foundations of statistical thermodynamics in the nineteenth century: we can make stunningly accurate predictions about the general properties and evolution of a *macroscopic* process even when the underlying *microscopic* details are effectively unpredictable.

Remarkably, our examination of systems dynamics in this section has independently led us to this very same conclusion. “If physics has had the sense of realism to divorce itself from microdeterminism”, Weiss writes, “there seems to be no reason why the life sciences, faced with the fundamental similitude between the arguments for the renunciation of molecular microdeterminacy in both thermodynamics and systems dynamics, should not follow suit” and reject the idea that macrodeterminacy is necessarily “reducible to a stereotyped performance by a fixed array of pre-programmed micro-robots” (Weiss 1969a: 30). Microdeterminacy is generally not a good explanation for the macrodeterminacy that we observe in the form, function, and behaviour of the systems that make up the living world.

This has major consequences for how we explain complex biological processes, such as development. At the level of the individual that undergoes it, embryogenesis is remarkably regular, stable, and predictable. Upon fertilization, it proceeds reliably through a well-defined sequence of stages, from the establishment of body axes to the differentiation of tissues and organs, culminating in the emergence of a coherent species-typical form. Despite the enormous complexity of the process, and the extraordinary diversity of living forms, the unfolding of development—and, perhaps most impressively, its *outcome*—is strikingly robust and consistent. A fertilized frog egg reliably develops into a frog (as opposed to anything else) just as a fertilized elephant egg reliably develops into an elephant.

**Fig. 5:**
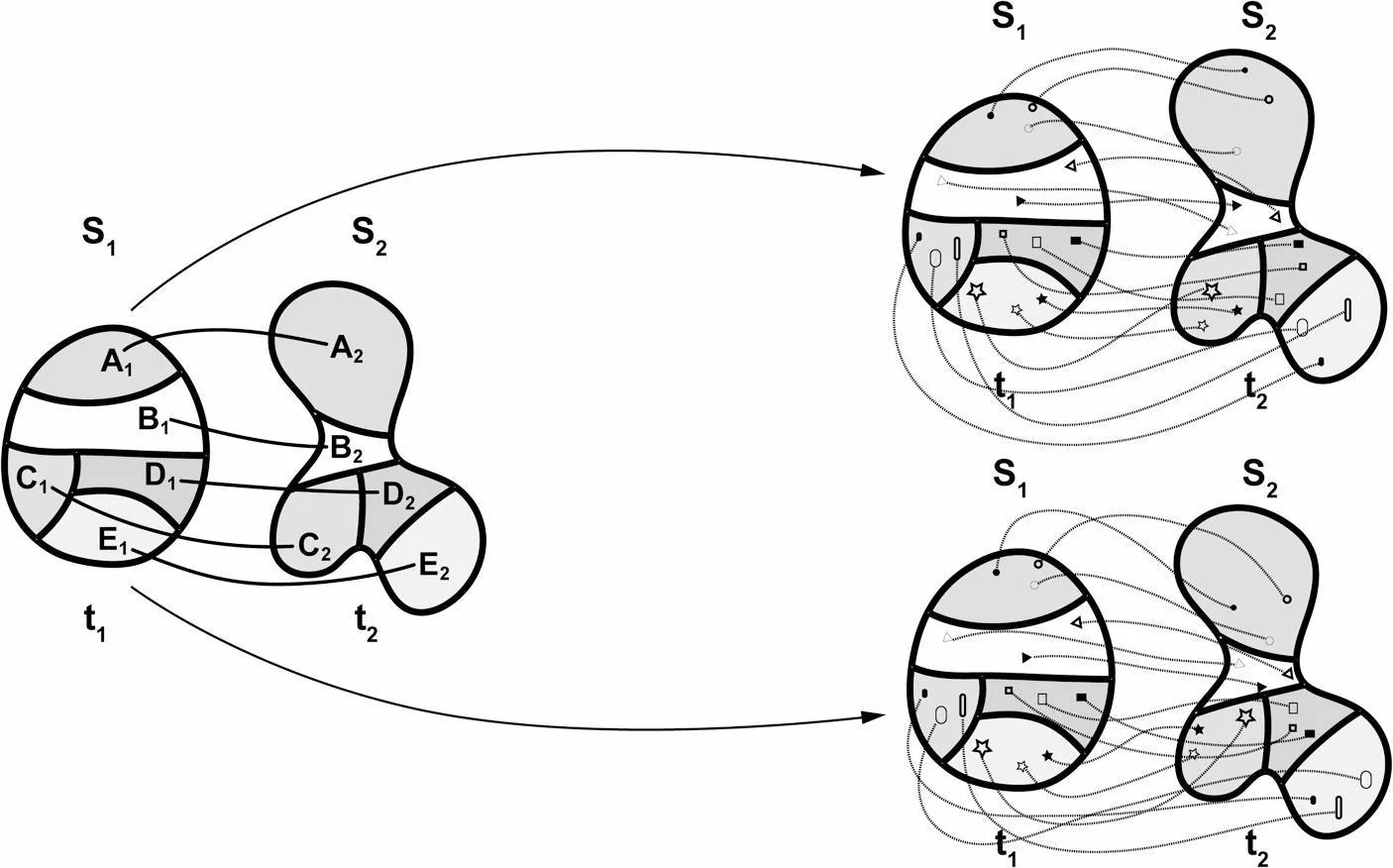
Schematic depiction of the persistence of the topological pattern of embryonic regions despite a lack of regularity in the positions of their constituent cells. The left side shows the *macroscopic view* of the regions of an embryo between developmental stages S_1_ and S_2_. At t_2_, every embryonic region (A_2_, B_2_, C_2_, D_2_, E_2_) can be traced back to a corresponding region (A_1_, B_1_, C_1_, D_1_, E_1_) at t_1_. Yet if we take a *microscopic view* of the same process at the same two points in time, shown on the right side, we find that even two very closely related embryos (top and bottom) differ drastically in the trajectories taken by the individual cells (depicted as symbols) in each embryonic region (redrawn from Weiss 1969a)

Now it might seem reasonable (again, prima facie) to suppose that embryogenesis is determined all the way down to the molecular level, but this is not in fact the case. What we find when we actually look is that there are innumerable ways to arrive to each developmental stage, and no two embryos ever undergo cellular differentiation in exactly the same way. Weiss illustrates this with an intriguing diagram that initially appeared in Weiss 1969a, and which I have redrawn in Fig. 5. It shows how the preservation of topological relations during two successive stages of development allows us to experimentally identify regions of an early embryo as being the predictably earmarked forerunners for the formation of specific organs (e.g., heart, brain, liver, kidneys), even though such clear-cut correlations between temporal stages do not hold at the cellular level (and even less so at the molecular level). In fact, when we follow the differentiation trajectories of particular cells in these prospective organ areas from the earlier to the later period, we find them taking far more fortuitous routes, diverging widely even between closely related embryos developing under the same conditions. Thus, although the ‘coarse-grained’ development of organ rudiments can be mapped out in a precisely predictable fashion, the ‘fine-grained’ molecular and cellular processes that underlie it remain unpredictable. The situation, Weiss observes, “is not unlike the formation of a river delta, of which one can only tell that the water will end up in the ocean, but never what precisely the distribution and configuration of the branches will be” (Weiss 1970a: 145).^30^

The macrodeterminacy of embryogenesis does not derive, then, from an underlying microscopic order, but arises *despite* the indeterminable microscopic processes that constitute it. There is, in other words, far less variance in the general unfolding of development than there is in the particular events that compose it, just as we noted in the system formula described above. This is yet another instantiation of the principle of “determinacy in the gross despite demonstrable indeterminacy in the small” (Weiss 1973: 21–22), which, Weiss insists, is a “prime feature of any system” (Weiss 1974: 49). Indeed, we should not forget that “the developmental system is a ‘system’” (Weiss 1971c: 39), and therefore everything we have discussed in this section concerning systems dynamics applies to it as well.^31^

Viewing development from a systems perspective allows us to see it in a new light, liberating us from the inevitability of the unabashedly deterministic understanding entailed by molecular biology, which construes it as “an unfolding of pre-existing capabilities, an acting-out of genetically encoded instructions” (Medawar 1965: 1329)—instructions that “carry an immensely complex, species-specific, regulatory program for the stepwise process of embryonic development” (Davidson 2012: R217).^32^ This deterministic understanding is increasingly being called into question as we are learning more about the indeterminacy that characterizes the molecular and cellular aspects of the process, which is largely due to the impact of *stochasticity* on gene expression (Elowitz et al. 2002; Raj and van Oudenaarden 2008) and on cell fate decisions (Losick and Desplan 2008; Balázsi et al. 2011).

The systems view suggests an alternative, *probabilistic* understanding of development, which stands in stark contrast to the prevailing deterministic one. Instead of grounding its determinacy from the bottom up by assuming that genes contain a program (i.e., a detailed set of step-by-step instructions that are precisely followed at the molecular level), it proposes instead that its determinacy is imposed from the top down by the developmental system as a whole, whose dynamics constrain and channel lower-level processes toward robustly reproducible phenotypic outcomes.

It also implies that focusing on the parts (i.e., genes and molecules) is the *wrong level of analysis*. We can illustrate this by returning to the analogy with statistical thermodynamics. One of the greatest discoveries of this field of physics was the realization, not just that statistics allows us to treat billions of molecules as a single ensemble—thereby avoiding the impractical task of examining each molecule individually—but, more importantly, that even if we *could* examine each molecule individually, this would not reveal anything that we could not already surmise from a statistical treatment of the ensemble as a whole. In other words, attending to the whole at the expense of ignoring the parts can be a strength rather than a limitation. Accordingly, we might find that focusing on the dynamics of the entire developmental system is a more productive strategy (given our explanatory goals) than insisting on examining “the detailed states and [differentiation] pathways of the components[, which] not only are so erratic as to defy definition, but even if a Laplacean spirit could trace them, would prove to be so unique and non-recurrent that they would be devoid of scientific interest” (Weiss 1969a: 12). This is, at any rate, one of the key lessons that the empirical findings symbolized in Fig. 5 allow us to draw.

The obstinate, quasi-ideological belief that only a genetic or molecular-level explanation of development will do is the reason for the proliferation of all that pernicious anthropomorphic terminology in the description of genes and molecules that we described in Sect. 4 as ‘molecular animism’. If we are to explain the distinctive developmental phenomena of coordination, control, integration, and robustness, we must raise our sights from the properties of individual genes and molecules to the collective dynamics of the developmental system ultimately responsible for them.

Of course, taking systems dynamics seriously does not entail denying the importance of genes in development—it simply helps us put them in their proper place. A developmental system possesses a certain ‘genetic endowment’ which provides ‘boundary conditions’ that shape its developmental trajectory, allowing sufficient latitude for non-genetic factors to jointly co-constitute the process (Weiss 1965: 33).^33^ This latitude affords the system the robustness required to ensure a reliable outcome despite inevitable environmental disturbances. Indeed, the unpredictable vicissitudes of the conditions in which development unfolds underscore just how unrealistic a deterministic view really is. Weiss illustrates this by pointing to the difference, when driving to an unfamiliar destination, between following a list of driving instructions and asking locals for directions along the way. Faced with unexpected contingencies, one is far more likely to get lost in the first scenario (Weiss 1970a: 124).^34^  

**Fig. 6:**
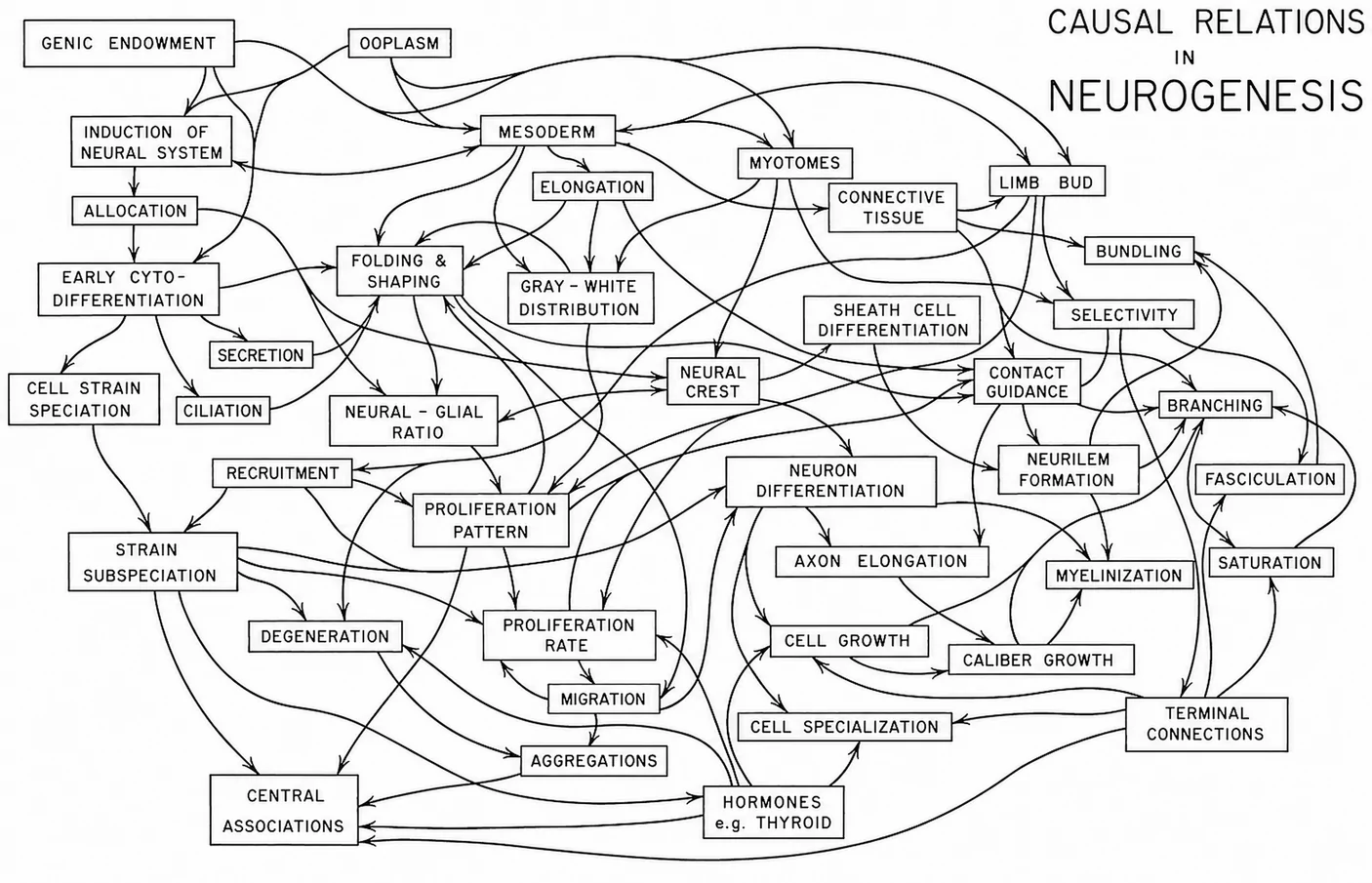
Network diagram showing the main tributary processes that lead from the fertilized egg with its dual endowment—genes embedded in ooplasm (top)—to the formation of the brain and spinal cord (bottom). The boxed-in titles are labels for the activities that have been singled out for analytical study. While the arrows reflect experimentally identified relations, as soon as we zoom out from these single causal links to the entire system, we are confronted with the integrity of the whole developmental fabric, in which ‘everything depends on everything else’ (after Weiss 1955b)

In place of a deterministic view of development as “the mechanical product of a bundle of linear [i.e., one-dimensional] ‘cause-effect’ chain reactions, reeling off in rigid sequence according to a minutely predesigned plan of clockwork precision” (ibid.: 96), a more plausible conception, Weiss suggests, is of a multi-dimensional *network* of numerous pathways that probabilistically converge toward a stable end result. Figure 6, first published in Weiss 1955b, offers an example of what Weiss has in mind.^35^ It is a flow chart of the major interrelated processes involved in the development of the nervous system, from its early stages (top) to maturity (bottom). Notice that as development progresses and differentiation and diversification increase, so does the number of systemic interconnections.

Despite being a “crude and incomplete” representation (Weiss 1955b: 393), the symbolic web of interlaced pathways shown in Fig. 6 helps convey the integrated nature of development. It shows why experimentalists hoping to find linear causal chains and strictly deterministic pathways are likely to be disappointed, regardless of the node in the network they choose to proceed from. Another virtue of this diagram is that it illustrates how the webbed topology of the process endows it with a considerable degree of elasticity and resilience in the way it unfolds—if any one branch line lags or fails, the remaining bypasses can still produce a viable, if slightly modified, result (Weiss 1966: 317–318, 1969a: 39).

As we bring our discussion of determinism to a close, it is a good moment to present another ‘system’ definition that we find in Weiss’s work, as it is one that captures the distinctive systems dynamics that have concerned us in this section:

Pragmatically defined, a system is a circumscribed complex of relatively bounded phenomena, which, within those bounds, retains a relatively stationary pattern of structure in space or of sequential configuration in time in spite of a high degree of variability in the details of distribution and interrelations among its constituent units of lower order. (Weiss 1973: 40)

What is particularly instructive about this definition, besides the fact that it emphasizes the constancy of the whole despite changes to the parts, is that it explicitly acknowledges that the characteristic invariance of a system is *temporal* as well as spatial. The retention of a system’s ‘relatively stationary pattern’ not only describes its ‘structure in space’ but also its ‘sequential configuration in time’.^36^ This is quite fitting, as the insights of systems dynamics we have articulated in this section apply to structures *and* to processes in equal measure—they are as relevant for describing the robustness and reliability of development as they are for capturing the dynamically stable organization of a cell or an organism.

With this, let us return one final time to the holistic maxim that we addressed in our examination of reductionism in Sect. 3. We concluded then that the best way to make sense of the idea that ‘the whole is more than the sum of its parts’ is to interpret the ‘more’ epistemically as referring to the greater amount of knowledge required to describe the behaviour of the parts when these are organized as an integrated unit. But perhaps we can now give the maxim an *ontological* interpretation as well. Our analysis of systems dynamics, as expressed mathematically in our system formula, revealed that the variance of a system as a whole—be it a cell, an organism, an ecosystem, development, or whatever—is much lower than the variance of its constituents put together. The delightfully ironic conclusion we arrive at is that, at least in one sense, what makes the whole *more* than the sum of its parts is that it varies *less* (and is therefore more orderly and regular) than would be suggested by examining the individual variances of all of its parts.

## Systems Thinking as an Antidote to Mechanicism

It should be clear by now that what systems thinking offers biologists, at a most basic level, is a *conceptual foundation* to ground their understanding of what an organism is. One might suppose that science can proceed in the absence of such foundations, through the mere accretion of empirical data, but this is an illusion. We may not be aware of them, or care very much to analyse them, but they are always there in the background, quietly shaping how we interpret and explain what we observe.^37^ The nature of the organism is a prime example. Biologists today seldom ask the question ‘what is an organism?’ explicitly, but that is because many already presuppose the answer: the organism is a machine.

The *machine conception of the organism* is the central tenet of *mechanicism*, a doctrine in biology with an illustrious pedigree that dates back to the rise of modern science in the seventeenth century.^38^ This conception, first embodied in the famous *bête-machine* of Descartes, evolved over the centuries as technology progressed, continuing to serve (more or less tacitly) as the conceptual foundation for how we think about organisms—not just physiologically, but also biochemically, developmentally, ethologically, and even evolutionarily. If it has not been explicitly defended all that often, it is only because it has tended to be taken for granted. However, the findings of modern biology are increasingly at odds with this conception,^39^ and here is where systems thinking can prove to be of most value, as the concept of a system, at least as Weiss understands it, is in many respects the *antithesis* of the concept of a machine.^40^

Recall that, as we pointed out in Sect. 3, it was Weiss’s rejection of the machine conception of the organism advanced by Loeb—the great arch-mechanist of the early twentieth century (Loeb 1912; see also Pauly 1987)—that prompted him to turn to systems thinking in the first place. The research he conducted for his PhD (on the response of butterflies to light and gravity) produced results that, in his own words, “proved totally incompatible with the mechanistic doctrines—or rather, rationalizations—of animal behavior prevailing at that time” (Weiss 1977: 17), which construed animals as little more than stimulus–response machines. “Right in my doctor’s thesis”, Weiss recounts, “I departed from antiquated mechanistic doctrine to introduce to the study of organisms the more flexible ‘system’ approach” (Weiss 1967a: 237). This desire to replace machine thinking with systems thinking in the conceptualization of organisms remained a central preoccupation for Weiss throughout his career. “Ever since 1925 [i.e., Weiss 1925]”, he asserted almost fifty years on, “I have presented the case for the dynamics of organisms as being in the nature of *systems*, rather than machine operations” (Weiss 1973: 11).

One could make the case that most of what we have discussed in the three preceding sections has been in the service of undermining the machine conception of the organism. The reason is that mechanicism, despite being the least familiar of our four ‘-isms’, is arguably the most fundamental, as it provides an ontological basis for the other three and thereby serves to legitimate them. It might be instructive, then, to begin our examination of mechanicism by making these connections explicit so that we can fully appreciate how the criticisms we have already provided help substantiate the claim that the organism is not a machine.

Mechanicism licenses *reductionism* because a machine is a composite entity *par excellence*; it is made up of clearly defined parts that interact to produce the behaviour of the whole. Accordingly, we can explain its workings by dismantling it and characterizing each of its components—and we can do so without irretrievably losing the properties of the whole. What makes reductionism so successful in the explanation of a machine (and, conversely, so limited in the explanation of a system) is that it involves reversing the process by which a machine is constructed. There is thus a perfect symmetry between the manner in which a machine is built (i.e., via the assembly of pre-existing parts) and the manner in which it is explained—a symmetry that is absent in the case of a system, such as an organism. In an organism, the existence of the parts does not precede that of the whole. The parts only acquire their respective identities *qua* parts as the whole progressively develops from an originally undifferentiated yet already integrated system. “The basic premise that embryonic development is an assembly process is false” (Weiss 1977: 26). Embryogenesis is a top-down process, whereas machine assembly is decidedly bottom-up.

Mechanicism also serves to justify *genocentrism*, especially the kind that seeks to establish the primacy and authority of genes by postulating the existence of a program encoded in the genome that controls the organism’s operations (Jacob and Monod 1961). The reason is that, as Jacob (1973: 9) readily acknowledges, the genetic program “is a model borrowed from electronic computers. It equates the genetic material of the egg with the magnetic tape of a computer”, adding that “everything urges one to compare the logic of heredity to that of a computer” (ibid.: 265). But this logic, which presupposes a clear separation of software and hardware, does not apply to systems. No part of a system is singly responsible for directing, controlling, or organizing the other parts. Recognizing that organisms are systems reveals the inadequacy of facile genocentric explanations, and it reminds us that lower-level phenomena cannot be properly accounted for if we overlook the higher-level systems that are ultimately responsible for them. Referencing again the essential contrast between systems and machines, Weiss (1969a: 13) clarifies that while “[i]n the system, the structure of the whole determines the operation of the parts; in the machine, the operation of the parts determines the outcome” (or behaviour) of the whole.

Mechanicism’s connection to *determinism* is perhaps the most evident, as machines have long been considered paradigmatically deterministic entities, given their impressive regularity and the exquisite ‘clockwork precision’ of their operation. We alluded to this in Sect. 5 when we indicated that a machine is deterministic ‘all the way down’, in the sense that the macrodeterminacy of its behaviour is the aggregative result of the microdeterminacy of all the mechanisms that make it up. A system is fundamentally different from a machine in this regard as well, as its macrodeterminacy does not depend on an underlying microdeterminacy at the level of its parts (we discussed this at length in the context of development). It is important to realize, however, that this general characteristic of systems dynamics does not mean that no biological phenomena can be explained as the outcome of ordered sequences of molecular steps (i.e., as instances of microdeterminacy). One of the chief motivations for “the adoption of the system concept in biological theory”, Weiss notes, “lies in the necessity of learning to *distinguish empirically* […] between phenomena calling for the probabilistic thinking of network theory on the one hand, and phenomena that are amenable to the strictly deterministic treatment of linear reaction chains, on the other” (Weiss 1971c: 18, my emphasis).

The great achievement of the molecular revolution of the last century was to have shown that many biological functions—DNA replication, cellular respiration, muscle contraction, neural reflex arcs, etc.—are in fact the product of intricate chains of tightly controlled molecular events proceeding in a regular fashion. It is for this reason that we can profitably describe them as *mechanisms*. But if organisms are systems rather than machines, then how do we account for the occurrence of mechanisms within them?

The answer lies in a feature of freely operating systems that we have not yet had the opportunity to discuss, which is that they exhibit a tendency toward progressive consolidation (Weiss 1970a: 118–127). We can illustrate this with the analogy of a flowing stream (cf. Nicholson 2018). Just as a water current that carves its own bed later becomes constrained by the channel it has created, when parts of a systemic network are repeatedly deployed to perform the same standardized operations, they become entrenched as pathways and acquire the recognizable character of mechanisms at the cost of losing their plasticity. This progressive consolidation can also be likened to the freezing of a stream. Evolution tends to ‘freeze’ as much free systemic activity as possible into fixed sequences of reactions (i.e., mechanisms) while also preserving sufficient freedom to allow the organism to respond adaptively to unpredictable changes in its internal and external environments.^41^

Accordingly, any highly conserved localized functions that are routinely described with the language of ‘control’, ‘regulation’, ‘compensation’, and so on, are better explained evolutionarily as specialized derivations of systems dynamics (i.e., from the top down), rather than mechanistically as automated operations of primary mechanisms expressing a genetic design (i.e., from the bottom up). The functions and behaviours that we identify as mechanisms are actually “very special cases and by no means prototypes of the far more fundamental and inclusive type of systems dynamics of organisms” (Weiss 1970a: 121).

It follows, then, that demonstrated biological instances of mechanistic microdeterminacy do not warrant the conclusion that a cell, or an organism, is a bundle of mechanisms. Structurally speaking, the pattern of a machine is a bit like a *mosaic* (or a jigsaw puzzle), in the sense that it can be dismantled and pieced together from its component mechanisms. The pattern of a system, in contrast, is more like a *spiderweb*, in the sense that the disruption of a single thread affects the structure of all the others. Nevertheless, parts of a system *can* acquire a quasi-mosaic state if the free dynamics of the whole becomes ‘frozen’—evolutionarily or developmentally—into a more consolidated condition. This is what allows us to identify them as mechanisms, which we can treat more or less autonomously depending on the explanatory context, even if they are ultimately expressions of the organization of the whole.^42^  

Beyond these interesting differences between the mechanisms we design in machines and the mechanisms we describe in organisms, a more serious problem with mechanicism is that it fails to capture the *processual *nature of the organism in the way that the system concept does. One of the most distinctive features of organisms is that they undergo continuous change. And not just gradual morphological changes over the course of their life cycles (let alone over longer, evolutionary timescales), but also continual biochemical changes as a consequence of metabolic turnover. The constant catabolic break down and anabolic buildup of materials that every organism experiences (mandated by the need to remain far from thermodynamic equilibrium) means that no organism is ever the same—materially speaking—from one moment to the next. Only in death, with the final cessation of metabolic activities and the inexorable move toward equilibrium, is the flux of life irrevocably terminated.

This ongoing material turnover takes place at every level of the biological hierarchy that we outlined earlier—indeed, it provides the *conditions* that secure the persistence of each hierarchical level. A population prevails because the organisms that are born replace those that die; an organism survives by continuously regenerating its organs and tissues; a tissue is maintained by the unremitting renewal of its cells; and a cell is energetically sustained by the incessant replenishment of its molecular content.

Notice that we already anticipated these processual features in our discussion of systems dynamics in Sect. 5, when we indicated that “the basic characteristic of a system is its essential *invariance* beyond the much more variant flux and fluctuations of its elements” (Weiss 1969a: 12). Our system formula also highlighted the significance of turnover, as it signals that a system retains its identity as a whole far more faithfully than the sum of its parts, which are relentlessly undergoing change and being exchanged. Likewise, the theoretical account we offered of the systems hierarchy, in which the stability of each level contrasts with the variability of the level below it, clearly mirrors the empirical one we have just described.

Machines do not display any of these characteristics. To be processual in the way a system is, Weiss writes, we need to imagine “a machine that not only builds itself but also keeps on reproducing spare parts for the replacement of worn-out units” (Weiss 1977: 27). In reality, a machine always consists of the same material parts, unless an external agent intervenes and replaces them. While the parts of a system at any given moment are only a temporary manifestation of the organizational dynamics of the whole, the parts of a machine remain firmly in place—the only real change they experience is eventual entropic decay. In a machine, the stability of the whole reflects the stability of its parts; in a system, it is maintained *despite* changes to its parts. Some machines are organized hierarchically like systems, but they form static hierarchies of structures. Systems form dynamic hierarchies of *processes* that are actively maintained at different timescales. It is because we are dealing with a hierarchy of nested processes in the living world that we find *downward* as well as upward causal interactions between the levels represented in Fig. 4.

Given that the turnover rate increases the further we go down the hierarchy, the processual nature of biological systems becomes more apparent as we move from the macroscopic to the microscopic scale. By the time we reach the cell, everything is quite evidently in flux. This is something that Weiss evocatively conveys in his molecular ecology concept, which emphasizes the dynamic interactions of the various molecular species in the cell, instead of assuming some rigid, mechanical chassis underlying it. Already in 1947, Weiss asserted that “the structural and working order of the cell is based not on the presence of a fixed mechanical framework pervading it—abundantly disproved by the facts—but on a regular distribution in space of the various intracellular processes” that constitute it (Weiss 1947: 255).

A major reason for the prevalence of the *machine conception of the cell* is that the methods and techniques traditionally used to probe the cell’s interior conceal the dynamic nature of its architecture, as researchers have to incapacitate it to render it visible (by fixing it, staining it, drying it, or whatever). The problem is that to study a cell frozen in time is already to approach it artificially as a static, machine-like object, rather than as the fluid system it is in reality. The structure of a machine, after all, can be grasped in abstraction from time (as it is not permanently changing), whereas the structure of a system cannot (systems, unlike machines, cannot be turned off). Using the snapshots produced by such methods—widely reproduced in textbooks—to infer the activity of a cell is as misleading as using still frames from a motion picture to infer the plot. One cannot tell whether a given snapshot portrays a momentary state of an ongoing process or a more permanent state of affairs. “Unless this ambiguity is fully appreciated and constantly borne in mind”, Weiss (1973: 39) warns us, “one runs the risk of mistaking the static picture of the cell for evidence of a mosaic of well-consolidated structures”.

Systems thinking in general, and molecular ecology in particular, allows us to “free the mental concept of the cell from the strait-jacket to which static textbook illustrations have committed it” (Weiss 1969a: 20). It encourages us to cultivate the mental habit of approaching subcellular structures as secondary derivatives, rather than as primary carriers, of cellular organization. Many such structures are indices or residues of the patterns of processes by which they have been formed—‘rhythmic structures’ produced by processes with periodicity. In the cell, “[d]ynamic process is the foundation of static form, rather than the reverse” (Weiss 1962: 24–25). What we perceive as static form is usually the product, transitory or lasting, of formative processes. As Weiss (1963: 393) pithily phrases it, “processed structure is but an outcome of structured processes”. Processes may not be visible in snapshots, but their structural effects certainly are, and systems thinking helps us interpret them.

In contrast to a machine, in which a fixed architecture performs predetermined functions, a cell is continuously transforming its own architecture (by modifying the balance between the inflow and outflow of matter) to keep up with its ever-changing functional demands. No machine organizes itself by autonomously exchanging its components so as to maintain its architecture in a dynamically stable state, yet that is precisely what happens in every cell. This is why there is no rigid structural frame underlying its form. The cytoskeleton is “a dynamic rather than static skeleton, maintained by metabolic energy” (Weiss 1947: 255). Unlike a machine, “the cell itself executes all its integrative and unitary systems functions without possessing anything resembling circuitry and wiring diagrams” (Weiss 1970a: 121).^43^  

Of course, the fact that cells are better understood as systems than as machines does not mean that the cell–machine analogy is wholly without merit. For instance, there are many respects in which a cell obviously resembles a *factory*: it is a highly organized, busy place that industriously manufactures numerous goods at various sites, processes and transports them to specific locations, and packages them with other products to be utilized, if not disposed of. Nevertheless, the usefulness of such comparisons is quite limited, and they can easily lead us astray if we take them too seriously. Weiss diagnoses the problems with this familiar analogy in a passage that deserves to be quoted in full, as it helpfully recapitulates many of the points we have made in the preceding discussion:

There is a major flaw […] in the analogy between a cell and a man-made factory. While in the latter, both building and machinery are permanent fixtures, established once and for all, many of the corresponding sub-units in the system of the cell are of ephemeral existence in the sense that they are continuously or periodically disassembled and rebuilt, yet always each according to its kind and standard pattern. In contrast to a machine, the cell interior is heaving and churning all the time; the positions of granules or other details in the picture, therefore, denote just momentary way stations […]. The only thing that remains predictable amidst the erratic stirring of the molecular population of the cytoplasm and its substructures is the overall pattern of dynamics which keeps the component activities in definable bounds of orderly restraints. These bounds again are not to be viewed as mechanically fixed structures, but as ‘boundary conditions’ set by the dynamics of the system as a whole. (Weiss 1969a: 21)^44^  

Overall, systems thinking helps bring into sharp focus many of the problems that stem from uncritically accepting the machine conception of the organism (and of the cell) as a foundation upon which to ground our understanding of living beings—which, of course, is not to deny its enormous heuristic value in certain contexts. And this has always been a big part of the difficulty, for the history of biology has shown us again and again how easy it is to mistake heuristic usefulness for ontological truthfulness. An idea need not be true to be useful (and vice versa). Richard Lewontin was fond of saying that “[t]he price of metaphor is eternal vigilance” (Lewontin 1963: 230), and this seems worth keeping in mind when reflecting on the almost irresistible appeal that the machine metaphor has for biologists.

This brings me to offer one last comment regarding the comparison between the machine and system conceptions of the organism, which is that it is not clear that in the latter case we are actually dealing with a metaphor applied to the biological domain. Weiss, for one, treats the system as an empirical concept—while it can be articulated abstractly (as we have done so at length in this paper), it designates entities that we *discover* in nature, rather than being a model devised in one domain (e.g., engineering) that we then *project* onto another (as is the case with the machine conception). “The existence of wholes [i.e., systems], in which parts take appointed places in mutual harmony consonant with the total pattern and task”, Weiss explains, “is an established fact of nature, not a privileged property and invention of the human mind” (Weiss 1960: 183).

In the final years of his career, Weiss became increasingly preoccupied with the metaphysical implications of the systems-oriented research program that he had pursued as an experimental biologist. He felt that recognizing the systemic character of organisms does not just prompt us to reject mechanicism; it also leads us to embrace *processualism* as the appropriate ontology for the living world. Here, once again, we find Weiss presciently anticipating contemporary developments—in this case, the recent ‘processual turn’ in the philosophy of biology.^45^ For Weiss, process ontology and systems thinking go hand in hand; one follows naturally from the other, as we have seen repeatedly in this section.

In his most overtly philosophical writings (e.g., Weiss 1970a, 1971c, 1977), Weiss steps back to consider how scientific inquiry is possible in a world that is fundamentally processual. Nature, he argues, appears to us initially as a vast, undifferentiated continuum. Yet we do not experience it simply as an undivided flow. As we attend to it, we pick out comparatively coherent configurations that persist, recur, or preserve a recognizable identity, distinguishing them from the more transient and irregular activities that recede into the background. These enduring configurations become the objects, events, and processes that we name and investigate. Science advances by conceptually delimiting these configurations within the wider continuum, treating them as distinct enough to be studied, and examining their properties and interactions. From this perspective, a system is not an isolated fragment of reality, but a relatively durable pattern identified within the larger continuum to which it remains dynamically embedded. The scientific practice of treating such patterns as discrete units of analysis has proven indispensable to their explanation, but this very success can also tempt us to regard them as self-contained entities and thereby overlook the continuous matrix of causal relations from which they were originally abstracted.

It seems fitting to end this discussion with what may well be the last definition of ‘system’ that Weiss offered in his published work, as it explicitly situates the concept within the broader picture we have just outlined:

a system is an empirical entity which in our experience has sufficiently durable identity to be defined on a macroscale as a whole, although from our knowledge of the parts alone into which we can physically or mentally fractionate the entity on the microscale, we could never retrieve the rule of order discernible in the intact macrosystem. (Weiss 1977: 55)

## Conclusions

This paper began with a widely acknowledged predicament. The extraordinary successes of molecular biology have transformed our understanding of cells and organisms, but they have also revealed the need for a complementary systems perspective. The new field of systems biology has generated powerful computational models, but it continues to approach the whole from the bottom up, as something to be reconstructed from comprehensive knowledge of the parts and their interactions. More formal approaches gathered under the rubric of systems theory can be illuminating, but they are unlikely to provide working biologists with the kind of intuitive, experimentally tractable concept of system that can guide them at the bench. As a result, the term ‘system’ remains conceptually thin: useful as a gesture toward complexity, organization, and integration, but too vague to do the explanatory work that biology now requires.

I have argued that we can begin to meet this need by recovering and updating the systems thinking of Paul Weiss—a figure barely familiar to historians and philosophers of biology, let alone experimental biologists,^46^ but whose writings allow us to articulate a fully-fledged system conception of the organism capable of doing real explanatory work. What sets our systems account apart from the more recent systems-theoretical proposals on offer is that it can be straightforwardly expressed in non-formal language, and instead of being imported into biology from other domains, it is derived empirically from experimental work. It also differs from current approaches in systems biology in that its starting point is not the list of parts and their interactions (from which the whole is to be pieced together somehow), but the integrated whole as such (whose parts can be identified and characterized by the roles they play in maintaining its overall dynamics). This reversal of perspective is crucial. It enables us to treat organization, closure, self-regulation, plasticity, dynamic stability, robustness, hierarchy, and many other properties of systems, not as recalcitrant phenomena that refuse to yield to molecular analysis, but as primary organismic features that can be elucidated and brought together under a single explanatory framework.

I have illustrated the *practical value* of systems thinking by deploying it against four deeply entrenched doctrines in modern biology. Against reductionism, the system concept shows that analytical decomposition necessarily eliminates information about the organization of the whole that cannot be recovered from an inventory of the parts. Against genocentrism, it shows that genes are indispensable participants in development and heredity, but not autonomous sources of organismic order; they function only within the organized cellular and developmental systems that make their activity possible. Against determinism, it shows that macroscopic regularity need not reflect rigid microscopic fixity, as it can also arise through the constraint of lower-level variability by higher-level organizational dynamics. And against mechanicism, it shows that organisms are not machines composed of pre-existing parts designed to perform externally assigned functions, but self-maintaining, processual wholes whose parts are continually produced, replaced, and coordinated by the systems to which they belong.

Of course, systems thinking is not the only route by which these four doctrines can be challenged. They have all been criticized before from other perspectives, most notably developmental systems theory (e.g., Oyama 1985; Oyama et al. 2001), the autonomy school of theoretical biology (e.g., Moreno and Mossio 2015; Mossio 2023), processual biology (e.g., Nicholson and Dupré 2018; Dupré 2021), and recent work on organismal agency (e.g., Walsh 2015; Fábregas-Tejeda et al. 2025). But Weiss’s older organicist view, besides brilliantly anticipating aspects of these newer perspectives, theoretically ties together its criticisms of the four doctrines by showing that their shortcomings, far from unrelated, are mutually reinforcing consequences of a deeply entrenched habit of thought: the tendency to atomize reality and to treat wholes (or systems) as derivative composites, rather than as primary, integrated units. The systems perspective developed in this paper reverses that priority; not by denying the importance of attending to the parts—be they molecules, genes, mechanisms, or whatever—but by situating them within the broader patterns of organization that make them biologically intelligible in the first place.

This is why non-formal theoretical analyses remain indispensable in biology. Mathematical models and computational techniques are unquestionably powerful tools, but they do not obviate the need for conceptual reflection. They cannot by themselves establish what a biological system is, adjudicate how its boundaries ought to be drawn, reveal the ways in which its parts depend on it, or determine what kind of explanation its organization requires. These are *conceptual* questions, though they matter greatly for experimental practice. A suitably articulated system concept—such as the one I have presented here—can serve not just a theoretical but also a *heuristic* function, shaping how biologists at the bench design experiments and interpret their results; for example, by directing their attention to what is preserved, what is disrupted, and what is irretrievably lost when a biological system is subjected to analytical decomposition.

Finally, the argument developed in this paper suggests a more general lesson about the relation between biology and its history. The present state of scientific knowledge is not the inevitable outcome of the past. Progress in science is not a linear accumulation of truths in which rejected ideas are discarded forever. Instead, it oscillates. The history of thinking about organisms, for instance, has moved through alternating waves of reductionistic enthusiasm and holistic correction. Ideas that were once neglected or prematurely abandoned later become newly relevant when the limitations of the dominant framework come into view. This is exactly what this paper has shown. Weiss’s system concept is arguably even more pertinent (and more needed) today than it ever was in his own lifetime. Similarly, his criticisms of molecular biology, as we have seen, have lost none of their bite, despite being more than fifty years old by now. If we are to advance the state of theoretical and philosophical debates in biology, it is our responsibility to learn about their past so that we do not inadvertently end up reinventing the wheel. Many more conceptual tools are likely to remain buried in history—just waiting to be dug up, dusted off, and put back to work.

## Data Availability

No datasets were generated or analysed during the current study.

## Footnotes

1 This is reflected in the descriptions that systems biologists give of their own field. Systems biology has been defined as “the study of the behavior of complex biological organization and processes *in terms of the molecular constituents*” (Kirschner 2005: 504, my emphasis) and as “a comprehensive quantitative analysis of the manner in which *all the components of a biological system* interact functionally over time” (Aderem 2005: 511, my emphasis). A prominent skeptic puts the point more bluntly: “systems biology sometimes appears to be little more than a new name for old-fashioned reductionist biology practised on an ever-larger scale, with ever-larger and more expensive machines” (Cornish-Bowden 2005: 317).

2 On the organicist movement, see Haraway 1976﻿, Nicholson and Gawne 2015, and Peterson 2016.

3 The biographical sketch offered in this section borrows liberally from other sources, especially Overton 1997 and Brauckmann 2004 and 2013, as well as from Weiss’s own scattered autobiographical recollections (e.g., Weiss 1967a). A book-length account of Weiss’s life and work remains to be written.

4 On the historical significance of the biological research conducted at the Vivarium, see Muller 2017.

5 As we will see later, it was in formulating his critique of Loeb’s ideas that Weiss first turned to systems thinking, which he continued developing until the end of his career (see Weiss 1977).

6 The rejection of Weiss by the Viennese academic community appears to have been partly motivated by antisemitism (see Taschwer 2019), but also by personal rivalries and “Weiss’ fondness for intellectual duels” (Brauckmann 2013: 2).

7 Weiss provides a detailed account of his rationale in Weiss 1953a; see also Appel 2000.

8 Although his system concept is Weiss’s most salient theoretical contribution, it is by no means the only one. Due to space restrictions, I will not be able to examine here another key notion that Weiss also deserves credit for helping to bring into biology: the concept of *field*. Weiss developed this notion out of his experimental work on regeneration, presenting it as a general concept for understanding morphogenesis in his early theoretical writings (Weiss 1926, 1928). He later gave it a much fuller treatment in *Principles of Development* (Weiss 1939). It would be interesting to see a study that does for Weiss’s field concept what this paper aims to do for his system concept.

9 For an excellent analysis of the connections between Weiss and Bertalanffy, see Drack et al. 2007.

10 In reality, the system concept in biology has even deeper historical roots, especially in the German tradition, where it can already be found at the turn of the twentieth century in the writings of Ewald Hering, Hans Driesch, and Nicolai Hartmann.

11 Interestingly, however, it is not exactly what Aristotle claimed. His original remark, found in Book VIII of the *Metaphysics*, is most accurately translated as “the totality is something *besides* the parts” (Aristotle 1984, *Met.* 1045a8–10, my emphasis).

12 Though if we interpreted Aristotle’s aphorism literally, we could conclude instead that ‘more is different’ (cf. Anderson 1972; Stumpf 2022).

13 It is ironic that just as revolutionary developments in modern biology have served to convince many of the omnipotence of reductionism as an explanatory principle, revolutionary developments in *modern physics*—especially statistical mechanics, quantum mechanics, and non-equilibrium thermodynamics—have served to disabuse many others of that very same conviction.

14 As the old nursery rhyme teaches us, ‘all the king’s horses and all the king’s men couldn’t put Humpty together again’.

15 Elsewhere, Weiss (1962: 43–44) offers yet another analogy, comparing the molecules in a cell with a laboratory full of bottled chemicals. The mere presence of the bottles is of no consequence. But if the bottles are opened and their contents are combined in the right proportions, predictable reaction products are obtained as the outcome of ‘organized behaviour’. By contrast, homogenization (Fig. 2b) is comparable to the violent smashing of the bottles and the resulting spilling and mixing of their contents.

16 This recognition of self-regulation, as with everything in biology, has numerous historical precedents. It can be found, for instance, in Gustav Fechner’s ‘principle of tendency toward stability’ and in Claude Bernard’s famous concept of the ‘internal milieu’. Another precursor, which Weiss credits as a formative influence on his systems thinking (as does Bertalanffy), is Wolfgang Köhler’s *Gestalt* theory, particularly its application to physical and biological phenomena (see Köhler 1920).

17 An instructive history of genocentrism can be found in Keller 2000; see also Nicholson 2025.

18 It is worth noting, though, that the concept of closure is interpreted in slightly different ways by these authors. Francisco Varela writes of ‘organizational closure’. For Robert Rosen, the central idea is ‘closure to efficient causation’. Stuart Kauffman emphasizes ‘catalytic closure’, whereas Álvaro Moreno and Matteo Mossio prefer to talk of ‘closure of constraints’.

19 Weiss’s analysis of the gene concept prefigures aspects of Lenny Moss’s critique in his book *What Genes Can’t Do* (2003).

20 Moreover, there are countless ways in which the function of BRCA1 can be potentially impaired. To date, more than *three thousand* (!) pathogenic variants of BRCA1 have been identified, each of which can be legitimately regarded as a ‘gene for breast cancer’.

21 As Weiss (1970a: 129) playfully remarks, “‘genes’ are not everything in life, even though nothing in life seems in the long run capable of proceeding without them. Water, for instance, is also indispensable for life, yet we no longer subscribe to the doctrine of Thales Miletus that water is the sole building block of the universe”.

22 Weiss was not the first to recognize or condemn this form of thinking, though. Before him, Ernest Everett Just (1939: 325) made the same point in a particularly vivid way: “Untutored savage man made his god as big as possible because his god could do everything. It remained for the geneticists to make one of molecular size, the gene. Here obviously infinite minuteness means infinite capacity. […] [T]he gene has almost magic power. This is physico-chemical biology with a vengeance!”

23 Weiss discusses the antithesis between preformation and epigenesis in Weiss 1940 and 1965.

24 Weiss’s depiction in Fig. 4 of the relations between levels in the hierarchical order of living systems predates by more than half a century the strikingly similar diagrams currently in vogue among systems biologists wanting to emphasize the complexity of biological causation and organization—see, e.g., the writings of Denis Noble (2006: 51, 2012: 59, 2016: 81).

25 We should not forget that Weiss himself was responsible for introducing the distinction between ‘molecular biology’ and ‘cellular biology’ in 1951—recall Table 1. When, a few years later, the former became the dominant force in biology, Weiss remarked with considerable surprise that “[w]hile I admit to my fatherhood of the term ‘molecular biology’ (coincidentally with Astbury [1952]) and unreservedly share the general admiration for the spectacular achievements and future prospects of work on that level, I merely meant the term to signify the lowest level in the total hierarchical structure of biological principles, without intending or expecting it to aspire to monopolistic status” (Weiss 1968﻿: xi).

26 Weiss hoped—in vain, as it turned out—that his molecular ecology model of the cell would be widely adopted and used to bridge the rapidly growing divide between molecular biology and cell biology. He ended his 1963 essay ‘The Cell as Unit’ by declaring that “the disciplined study of the *systemic* properties of the cell—of ‘*cell biology’*—that is, the manner in which its molecular components, which are the prime objects of ‘*molecular* biology’, are subordinated to ordered group coexistence in a system of ‘molecular ecology’—is one of the major challenges and tasks of modern science. To meet it, we must face it. To face it, we must see it. To see it, we may even at times have to put on blinders so as to reduce the dimming effect of contrast engendered by all the brilliant light that emanates from ‘molecular biology’” (Weiss 1963: 397). Interestingly, the molecular ecology metaphor has started to resurface in the last few years. For a recent appraisal, see Nathan 2014.

27 We will continue discussing the biological significance of the material turnover of systems in Sect. 6.

28 This recurring motif in the hierarchical organization of the living world helps explain why Weiss considers it so instructive to view the relation between a cell and its constituent molecules through the lens of the relation between a population (or an ecosystem) and its constituent organisms, as his concept of molecular ecology invites us to do.

29 Weiss prefers the terms ‘determinacy’ and ‘determinable’ over the more abstract philosophical concept of determinism because he takes them to be of empirical origin, and to refer to the predictability of concrete processes. These terminological choices matter to Weiss because he insists on basing his theoretical proposals on experimental findings rather than on philosophical speculation.

30 It is worth adding that as a result of the progress made in experimentally mapping out developmental cell fates since Weiss’s time, for very simple multicellular organisms such as the tiny nematode worm *Caenorhabditis elegans* we can actually track the individual lineages of all 671 of its embryonic cells (Sulston et al. 1983). But Weiss’s general point stands, as such perfectly predictable behaviour is the exception rather than the rule—it remains a practical impossibility for most other developmental systems due to the far greater compounding effect of the ‘noise’ produced by stochastic fluctuations.

31 Notice that because of the fact that what Fig. 5 shows (i.e., the conservation of order in the whole despite unpredictable variation in the parts) reflects a property of systems at all hierarchical levels, the figure lends itself to different interpretations depending on the scale. At the level of the embryo, the tiny symbols on the right side of the figure stand for cells. But at the level of the *cell*, they stand for *molecules*. When interpreted in this way, Fig. 5 takes on a completely new meaning, illustrating, not a developing embryo, but the internal dynamics of a cell over time (in accordance with Weiss’s own molecular ecology model).

32 There is nothing particularly modern about deterministic views of development. In the mid-seventeenth century, René Descartes had already remarked that “if one had a good knowledge of all the parts of the seed of some species of a particular animal, man for example, one could deduce from this alone, by entirely certain and mathematical arguments, every shape and structure of each of its bodily parts” (Descartes 1998: 200).

33 Weiss’s views on the role of genes in development accord well with those of his fellow organicist Conrad Hal Waddington, exemplified in his famous epigenetic landscape model, and whose work Weiss cites approvingly (e.g., Weiss 1969a: 38).

34 John Dupré, writing 35 years later, offers another everyday analogy to make the same point about the non-deterministic nature of development: “If I ask someone to go to the shop and buy me a loaf of bread, and they agree, I am fairly confident that the outcome will be as I intend. If I provide a deterministic programme—take 12 paces north-west, raise hand, turn knob, push, etc., there are too many unanticipated interventions that can derail the process for me to have much hope of success” (Dupré 2005: 206).

35 As with some of his other figures, Weiss reuses this one often in later writings (e.g., Weiss 1966, 1969a, 1971c, 1977, 1978).

36 This points to another unappreciated parallel with Waddington, who coined the term ‘homeorhesis’ as a dynamic counterpart to Walter Cannon’s homeostasis in order to describe the relative invariance, not of a physiological state, but of a developmental *trajectory* in the face of perturbations (see Waddington 1957).

37 In making this point, I am reminded of Daniel Dennett’s famous quip that “there is no such thing as philosophy-free science; there is only science whose philosophical baggage is taken on board without examination” (Dennett 1995: 21).

38 I am using the less familiar word ‘mechanicism’ to terminologically distinguish this doctrine from other meanings that the concept of mechanism has acquired in biology. A detailed historical and philosophical analysis of this concept can be found in Nicholson 2012.

39 For a comprehensive critique of the machine conception of the organism, see Nicholson 2013, 2014b, 2018, 2019﻿, and 2020.

40 Not just Weiss, in fact. Bertalanffy—the other great organicist systems thinker from Vienna—likewise formulated his own systems theory of life in direct opposition to the reigning machine conception of the organism (see, e.g., Bertalanffy 1952).

41 Weiss also uses the flowing stream analogy to describe what happens during development: the differentiation of cells in an embryo reflects the gradual ‘solidification’ of an initially ‘fluid’ equipotential dynamic state. As the embryo develops, its parts behave less like systems and more like *mechanisms* (Weiss 1970a: 122). Two decades earlier, Bertalanffy had made exactly the same point: “Progressive differentiation means, at the same time, progressive *mechanization*” (Bertalanffy 1952: 46).

42 This systems-oriented conception of biological mechanisms is very different from the orthodox accounts that philosophers have given of this concept in recent decades (notably Machamer et al. 2000; Glennan 2002; Bechtel and Abrahamsen 2005). The contemporary philosophical accounts that come closest to Weiss’s notion are those that emphasize the pragmatic and contextual character of mechanism-talk in biology (e.g., Nicholson 2012; Moss 2012; Dupré 2013; Ioannidis and Psillos 2022).

43 It is remarkable to find biologists today increasingly discussing the cell’s architecture in precisely these terms. Consider the following remark, taken from the abstract of a recent scientific review: “Unlike the engineered macroscopic structures that we commonly build, biological structures are dynamic and self-organize: they sculpt themselves and change their own architecture, and they have structural building blocks that […] constantly come on and off. A description of such structures defies current traditional mechanical frameworks” (Dumont and Prakash 2014: 3461). For a detailed examination of these fascinating developments, see Nicholson 2019 and 2020.

44 Although we have focused on the cell here, that is not the only entity (or level) that Weiss examines from a non-mechanical, processual perspective. For example, in a paper titled ‘Panta’ Rhei—And So Flow Our Nerves’ (1969b), Weiss emphasizes the fluid nature of *nerves*, and rejects the (at the time, popular) analogy between the *nervous system* and a telephone network.

45 The case for adopting a process ontology in biology has been put forward systematically in Dupré and Nicholson 2018. For additional arguments in support of this position, see the seventeen other essays in Nicholson and Dupré 2018.

46 There are exceptions, of course. Scott Gilbert is a prominent developmental biologist who has long recognized the theoretical value of Weiss’s ideas (e.g., Gilbert et al. 1996; Gilbert and Sarkar 2000; Gilbert 2014: 627–634). A more recent example is the Laboratory of Morphogenesis co-led by Amy Shyer and Alan Rodrigues at Rockefeller University, which is explicitly guided by Weiss’s conceptual approach to biological problems; see: https://shyerrodrigueslab.com.
